# Interplay Between Exfoliation and Functionalization Strategies for Group VI Layered Transition Metal Dichalcogenide Dispersions

**DOI:** 10.3390/nano16070429

**Published:** 2026-03-31

**Authors:** Quoc Minh Tran, Pailinrut Chinwangso, Minh Dang Nguyen, Supawitch Hoijang, Melissa Ariza Gonzalez, Ruwanthi Amarasekara, Ramtin Yarinia, Yunsoo Choi, T. Randall Lee

**Affiliations:** Department of Chemistry and the Texas Center for Superconductivity, University of Houston, 4800 Calhoun Road, Houston, TX 77204-5003, USA; qmtran20@cougarnet.uh.edu (Q.M.T.); pchinwa3@central.uh.edu (P.C.); mdnguy27@cougarnet.uh.edu (M.D.N.); marizago@cougarnet.uh.edu (M.A.G.); rhmamara@cougarnet.uh.edu (R.A.); ryarinia@cougarnet.uh.edu (R.Y.); ychoi3@cougarnet.uh.edu (Y.C.)

**Keywords:** transition metal dichalcogenides, solution-processing exfoliation, colloids, surface functionalization

## Abstract

The ability to efficiently tailor the surface properties of layered transition metal dichalcogenide (LTMD) dispersions is critical for optimizing performance and enabling scalable manufacturing techniques, such as spray coating and inkjet printing, for optoelectronic, energy storage, and sensing applications. Group VI LTMDs, owing to their unique properties in the monolayer architecture, offer exceptional potential; however, the properties of exfoliated dispersions are strongly dependent on the specific solution-processing techniques employed. These techniques determine the choice of subsequent surface functionalization strategies and, consequently, the characteristics of the resulting functionalized hybrids. Furthermore, the inherent heterogeneity of solution-processed dispersions—manifested, among other factors, in broad distributions of flake thickness and lateral size—remains a significant challenge and strongly influences the behavior of hybridized materials. As a result, exfoliation-method-dependent properties and dispersion heterogeneity introduce substantial complexity in the selection of appropriate surface-tailoring strategies, characterization methodologies, and data interpretation. To address these challenges, we systematically classify exfoliated Group VI LTMD dispersions according to their exfoliation methods and highlight recent findings that challenge previously accepted assumptions in the field. Finally, we provide perspectives on surface functionalization approaches for Group VI LTMDs and discuss key limitations associated with the characterization of these newly hybridized materials.

## 1. Introduction

Layered transition metal dichalcogenides (LTMDs) constitute a family of two-dimensional (2D) materials with unique van der Waals (vdW) crystal structures. They follow the general chemical formula MX_2_, where M denotes a transition metal and X represents a chalcogen atom (X = S, Se, or Te). Among this class, Group VI LTMDs have attracted substantial research interest owing to their chemical stability, mechanical flexibility, and semiconducting behavior, which become particularly pronounced at the monolayer level [1]. These attributes have positioned Group VI LTMDs as promising materials for applications in electronics, optoelectronics, spintronics, magnetics, catalysis, and energy storage [2]. Achieving many of these applications requires the synthesis of high-quality, large-scale LTMD thin films on diverse substrates, which is most commonly accomplished using chemical vapor deposition (CVD) techniques [3,4].

Recently, solution-based methodologies have emerged as cost-effective and scalable approaches for producing few-layer to monolayer colloidal dispersions of Group VI LTMDs. The ability to form stable dispersions enables the formulation of ink materials suitable for batch processing, facilitating their implementation in deposition techniques such as inkjet printing and roll-to-roll fabrication. This approach holds significant potential for large-area fabrication and integration into advanced technologies, including energy generation and storage [5], field-effect transistors [6], memristors [7], optoelectronic devices [8], sensors [9], and filters [10], among others. However, such deposition techniques typically yield thin films composed of restacked multilayer LTMDs, thereby compromising the distinctive properties of isolated LTMD monolayers. Furthermore, precise modulation of interfacial properties, as well as the electronic and optoelectronic characteristics of LTMD dispersions, is crucial for achieving targeted functionalities [11,12]. Consequently, surface functionalization with organic ligands, leading to hybridized materials, has emerged as a powerful strategy to address these limitations by enabling control over material properties and performance [13].

In the case of Group VI LTMD dispersions, several persistent challenges remain that limit the development of functionalization strategies and hinder comprehensive characterization and understanding of these systems, including the following:

(1) Natural Group VI LTMDs exhibit semiconducting characteristics and predominantly crystallize in the 2H phase (or 1H at the monolayer level). Owing to their fully occupied valence band, 1/2H LTMDs are chemically inert, particularly on the basal plane, which is passivated by the electron density associated with the lone pairs of chalcogen atoms.
(2) Effective tailoring of interfacial properties and tuning of the electronic and optoelectronic characteristics of LTMD dispersions generally requires interactions beyond physisorption, which are typically achieved through basal-plane functionalization. Due to the intrinsic chemical inertness of the basal plane, such strategies often involve trade-offs in crystallographic integrity, including phase transformation from the semiconducting 1/2H phase to the metallic 1T phase, which leads to the loss of semiconducting properties.
(3) The properties of exfoliated dispersions vary significantly depending on multiple factors, including exfoliation methods, solvents, and starting materials. These parameters therefore play a critical role in determining both the feasibility of surface functionalization strategies and the properties of the resulting hybridized materials.
(4) Heterogeneity in phase composition, flake size, thickness, and surface chemistry complicates characterization and, consequently, the realization of uniform functionalization.

Given these challenges, this perspective aims to explore the interplay between exfoliation and functionalization and how this relationship influences the final physicochemical properties of solution-processed hybridized Group VI LTMDs. It discusses unavoidable challenges arising from dispersion heterogeneity and illustrates how fractionation techniques reveal property variations within a single hybridized system. Technical considerations related to sample preparation and characterization are re-examined, highlighting that widely accepted interpretations, although supported by experimental observations, might not universally apply across all systems.

Finally, based on recent findings, we emphasize that significant gaps remain in the exploration of organic ligands used to functionalize specific classes of exfoliated Group VI LTMD dispersions. These gaps may manifest in ways that contradict common assumptions in the field. To address this issue, we classify exfoliated Group VI LTMD dispersions according to key structural and electronic features and tentatively propose prospective functionalization strategies guided by principles of nucleophile–electrophile interactions and Lewis acid–base chemistry. This perspective is intended to provide researchers from diverse scientific backgrounds—including materials science, nanoscience, and chemistry—with a conceptual framework for optimizing the properties of hybridized LTMD materials for targeted applications, which are often complicated by the inherently heterogeneous nature of solution-processed dispersions.

## 2. Structure–Property Correlations in Group VI LTMDS

Group VI LTMDs exhibit a layered structure in which a monolayer consists of a central atomic layer of transition metal atoms (M = Mo or W) sandwiched between two atomic layers of chalcogen atoms (X = S, Se, or Te) [1]. These monolayers naturally stack to maximize interlayer vdW interactions, forming multilayer structures. The weak vdW interactions between adjacent layers enable exfoliation of bulk materials into monolayers through the application of external forces [14]. Within an individual layer, atoms are strongly bonded via covalent interactions, and the typical monolayer thickness ranges from 6 to 7 Å [15].

**Figure 1:**
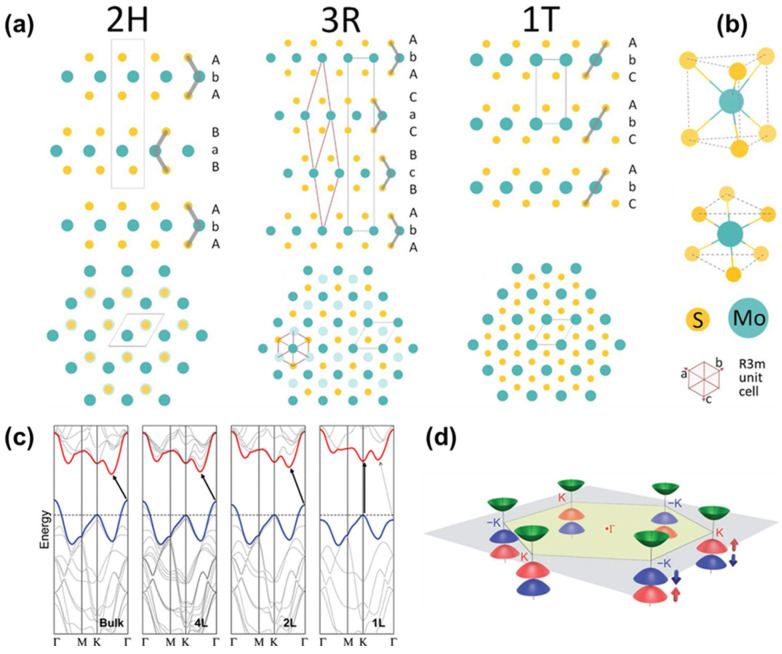
(**a**) Three representative polymorphs of Group VI LTMDs arising from different stacking sequences of single layers, shown in side view (top) and top view (bottom), and (**b**) the corresponding Mo–S coordination geometries: trigonal prismatic coordination for the 1/2H and 3R phases (top) and octahedral coordination for the 1T phase (bottom). Reprinted in part with permission from Ref. [42]. Copyright 2021, American Chemical Society. (**c**) Energy band structures of molybdenum disulfide (MoS_2_) in bulk, four-layer, bilayer, and monolayer forms. In the monolayer limit, the excitonic transition is direct, whereas thicker structures exhibit an indirect bandgap. Reprinted in part with permission from Ref. [27]. Copyright 2010, American Chemical Society. (**d**) Schematic illustration of the valley spin–orbit coupling phenomenon in monolayer MoS_2_. Reprinted in part with permission from Ref. [25]. Copyright 2012, American Physical Society.

The lattice structures of LTMDs are primarily determined by the coordination geometry between M and X atoms, as well as by the stacking sequence of the monolayers. Group VI LTMDs exhibit three principal polymorphs: (1) hexagonal phase, denoted as 1H for monolayers and 2H for multilayers, featuring trigonal prismatic coordination of the metal center with *D*_3*h*_ symmetry; (2) the rhombohedral (3R) phase, which also exhibits trigonal prismatic coordination and *D*_3*h*_ symmetry; and (3) the tetragonal (1T) phase, characterized by octahedral coordination of the metal center with *O_h_* symmetry (Figure 1a,b). The numerical designation reflects either the number of layers per stacking sequence or the number of transition metal atoms per unit cell, while the letter denotes the crystal lattice systems. For example, the 2H phase adopts an ABAB stacking sequence, whereas the 3R phase follows an ABCABC sequence, both retaining trigonal prismatic coordination. In contrast, the 1T phase exhibits an AAAA stacking arrangement. In some cases, the 1T phase undergoes lattice distortion, giving rise to a lower-symmetry 1T′ phase [16]. This distortion is commonly associated with Jahn–Teller effects, leading to concomitant structural and electronic modulation [17].

The lattice structure of LTMDs is closely related to their electronic properties, which are governed by the progressive filling of electrons into the non-bonding d bands located between the bonding and antibonding M–X states [1]. In materials where these bands are partially filled, such as Group V LTMDs, metallic behavior is observed. In contrast, Group VI LTMDs possess fully occupied non-bonding d bands, leading to a bandgap and semiconducting behavior. Natural Group VI LTMDs predominantly adopt the thermodynamically stable 1/2H semiconducting phase. When excess electrons are introduced and populate the conduction band, a crystallographic transformation might occur, inducing a phase transition from the 1/2H phase to the 1T phase [16,18]. The 1T phase is metastable and can readily revert to the 1/2H phase through thermal annealing or other external stimuli [19,20,21]. Charge injection into the conduction band significantly enhances material reactivity by increasing electron density and inducing a negatively charged surface. Nevertheless, an optimal range of electron injection exists in which no crystallographic phase transition occurs, allowing the material to retain its semiconducting character while exhibiting increased electron density [22,23].

The most distinctive properties of Group VI LMTDs arise from their thickness-dependent behavior, which is governed by band-structure modifications induced by quantum confinement effects (Figure 1c,d) [24,25]. At the monolayer limit, the bandgap transitions from indirect to direct, enabling the emergence of photoluminescence (PL), which is typically absent in bulk counterparts [24,26,27]. Numerous studies have demonstrated that the electronic and optical properties of single-layer LTMDs can be effectively tuned by applied strain [28,29,30] or relatively weak physisorption through mechanisms such as dielectric screening [31,32,33], dipolar interactions [34,35,36,37], and charge transfer [38,39,40]. This tunability highlights the versatility of Group VI LTMDs for optoelectronic and photonic applications [41]. However, strategies that are effective for well-defined single-layer LTMDs might not necessarily exhibit the same efficacy in colloidal systems, which involve multiple interacting components. An additional factor that must be considered is the unavoidable presence of defects in LTMD materials. Owing to the complexity and diversity of defects—including vacancies, grain boundaries, and dislocations—and the additional properties they impart, this perspective primarily considers defects as chemically active sites during functionalization.

## 3. Influence of Exfoliation Techniques on the Characteristics of LTMD Dispersions

The exfoliation techniques used to produce LTMD dispersions play a critical role in defining their initial physicochemical characteristics and, consequently, the feasibility and implementation of subsequent surface hybridization strategies. Although numerous studies have reported exfoliation methods capable of producing high-quality, scalable, batch-processed few- to monolayer LTMD dispersions, comparatively little attention has been devoted to the diversity of properties exhibited by the resulting dispersions. This section focuses on this aspect and systematically classifies exfoliated Group VI LTMD dispersions according to their intrinsic characteristics.

### 3.1. Intercalation-Driven Exfoliation

Effective exfoliation requires overcoming the adhesion force, namely the interlayer vdW interactions, between adjacent layers. In intercalation-driven exfoliation, guest species penetrate the interlayer galleries of LTMDs, triggering post-intercalation effects that promote delamination. These effects include an increase in interlayer spacing [43], abrupt gas evolution [44,45,46,47], and energetically favorable solvation processes [48].

The first experimental attempts to obtain few- to monolayer dispersions of Group VI LTMDs employed a chemical pathway based on alkali cation intercalation [44,45,46]. For instance, a strong alkali metal-based reductant, such as *n*-butyllithium (*n*-BuLi), was used to partially reduce LTMDs, imparting a high negative charge density to the layers. To compensate for this excess charge, alkali cations intercalate into the interlayer galleries, forming intercalated hybrid structures. Subsequent exposure of these hybrids to water induces solvation of the alkali cations and generates hydrogen gas, leading to spontaneous delamination. The resulting exfoliated 2D flakes can be redispersed in suitable solvents with brief sonication.

The nature of the intercalated hybrids and the resulting exfoliated 2D flakes warrants further consideration. *N*-BuLi, as a strong reducing agent, donates electrons to the unoccupied conduction band of LTMDs, inducing a phase transition. The intercalated hybrids adopt the 1T lattice structure, which is stabilized by charge neutrality through cationic intercalants. Removal of the cations does not revert the exfoliated materials to the original 1/2H phase; instead, the structural and electronic characteristics of the 1T phase are retained [49]. Consequently, the resulting LTMD dispersions exhibit three defining features: (1) loss of semiconducting behavior, (2) high electron density, and (3) a negatively charged surface. In addition to *n*-BuLi, other organolithium reagents, including lithium borohydride (LiBH_4_), naphthalene lithium (Nap–Li), and pyrene lithium (Py–Li), have also been employed. However, caution is required, as certain reagents can induce irreversible side reactions and chemical decomposition of LTMDs [50,51,52]. Herein, we note that exfoliation following wet-intercalation-chemistry has long faced the challenge of an incomplete transition from the 1/2H phase to the 1T phase. This issue has been addressed only recently with the report of nearly pure-phase metallic MoS_2_, which enables the preparation of homogenous system composed of exclusively of the metallic 1T LTMDs [53].

In solid thin films, a gradual transition from the 1T phase to 1/2H phase has been observed over extended periods under ambient conditions. This transition is attributed to oxidation processes that effectively remove excess electrons from the 1T phase. In contrast, the 1T phase can be stabilized in colloidal dispersions using appropriate solvents. Accelerated phase transformation in solid films generally requires thermal treatment [19] or external stimuli such as laser irradiation [21]. An alternative approach involves heat treatment in solution, which offers greater flexibility for subsequent fabrication steps [20]. In all cases, processing must be conducted under oxygen-free conditions, as LTMDs are susceptible to oxidation at elevated temperatures.

**Figure 2:**
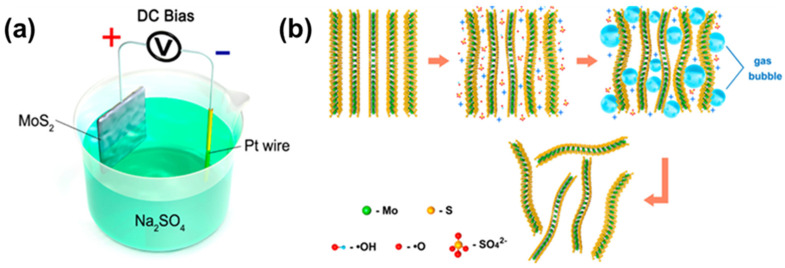
(**a**) Schematic illustration of the experimental setup for electrochemical exfoliation of single-crystal MoS_2_ via anodic intercalation, and (**b**) the corresponding exfoliation mechanism. Reprinted in part with permission from Ref. [47]. Copyright 2014, American Chemical Society.

To mitigate the degradation of crystallographic integrity, precise control over the extent of electron donation into LTMDs is essential. This has been demonstrated by regulating the stoichiometric ratios of reactants [22]. Alternatively, electrochemical methods allow fine control over electron injection through adjustment of applied voltage, intercalation time, and the nature of the intercalants, enabling the production of large-area monolayer flakes with lateral dimensions approaching the micrometer scale. For example, sulfate (SO_4_^2−^) anions can be electrochemically intercalated into LTMDs under an applied positive voltage (Figure 2a) [47]. During this process, water oxidation generates •OH and •O radicals, which co-intercalate with SO_4_^2−^ anions into the interlayer galleries (Figure 2b). Subsequent oxidative reactions release O_2_ or SO_2_ gas, further expanding the interlayer spacing of the host lattice. Few- to monolayer flakes can then be obtained through brief sonication.

Recent studies have revisited superlattice structures based on Group VI LTMD host frameworks, demonstrating that bulky organic quaternary ammonium salts can be effectively intercalated into the interlayer galleries. In these systems, the vdW dimensions of the intercalants play a decisive role in regulating the extent of electron injection through a charge-balance mechanism [54,55,56]. For instance, electrochemically induced intercalation of cetyltrimethylammonium (CTA^+^) cations from cetyltrimethylammonium bromide (CTAB) solutions typically induces a phase transition in single-crystal 2H-MoS_2_, yielding the metallic 1T phase [55]. In contrast, intercalation of bulkier tetraheptylammonium (THA^+^) cations from tetraheptylammonium bromide (THAB) solutions preserves the semiconducting nature of single-crystal 2H-MoS_2_ [55].

Whether intercalation is driven by reductive (cathode intercalation) or oxidative (anode intercalation) processes, Group VI LTMDs undergo a certain degree of structural damage. Excessive reduction can lead to irreversible chemical transformations, including, in some cases, the formation of metallic Mo and Li_2_S [50]. In addition to crystallographic alterations, reduction also induces defects, such as the removal of chalcogen atoms at edge sites or the creation of vacancies on the basal plane. Conversely, oxidation generally induces a higher degree of defects, as highly reactive oxidants (e.g., radicals, applied positive voltages, and water) promote degradation of LTMD structures [57].

In summary, several key aspects related to the properties of Group VI LTMD dispersions obtained via intercalation-driven exfoliation methods can be identified as follows:

(1) Strong reducing agents typically induce crystallographic phase transformation, accompanied by intercalation of guest species to compensate for excess charge. The surfaces of exfoliated metallic flakes are negatively charged, rendering them chemically reactive.
(2) Milder electron-donation strategies preserve the semiconducting phase of LTMDs; however, a negative surface charge is still expected.
(3) Regardless of the intercalation strategies, LTMDs undergo a certain degree of structural damage.

### 3.2. Surface Matching–Sonication-Mediated Exfoliation

An alternative exfoliation approach is liquid-phase exfoliation (LPE), first proposed by Coleman and co-workers in 2011 [58]. This technique involves a two-step process: first, the weak interlayer interactions are overcome by supplying sufficient energy to induce exfoliation; second, the resulting 2D flakes are stabilized by solvent molecules or surfactants, which inhibit restacking and aggregation.

The primary advantage of LPE lies in its ability to preserve the crystallographic integrity of the 1/2H phases of Group VI LTMDs while avoiding additional chemical reactions that might generate undesired by-products. However, several limitations should be noted:

(1) Solvent selection is constrained by the surface–solvent matching mechanism [58,59,60,61,62,63].
(2) To expand the range of usable solvents, particularly aqueous systems, surfactants are typically required [64,65,66,67]. The surface properties of the resulting flakes are therefore dictated by the surfactants, which cannot be completely removed [64,67,68,69].
(3) Flake scissoring and defect formation are inherent challenges associated with the processing techniques. For example, prolonged exposure to high-power probe sonication yields smaller flakes [70] compared to low-power shear mixing [64] or bath sonication [58].

In addition to the approaches discussed above, several methods incorporate a pre-treatment step to facilitate exfoliation, including ball milling, grinding, salt-assisted techniques, and gas pre-expansion [71,72,73,74,75,76]. Following pre-treatment, the materials are typically subjected to sonication to induce delamination.

### 3.3. Microwave-Assisted Exfoliation

Microwave-assisted exfoliation involves an initial pre-wetting step, in which LTMDs are treated with compatible solvents, followed by microware irradiation. It is proposed that these solvents intercalate into the interlayer galleries of LTMDs. Upon microwave absorption, these solvents undergo rapid evaporation, generating internal pressure that facilitates layer separation [77]. In certain cases, dipolar organic solvents or ionic liquids, which exhibit strong electromagnetic absorption, undergo oscillatory motion under microwave irradiation, thereby initiating exfoliation from edge sites [78]. This approach has demonstrated the ability to produce exfoliated flakes with minimal structural defects while achieving relatively large lateral dimensions (1–4 μm) [77,78]. The primary factor governing the properties of the resulting dispersions is the choice of dispersing solvent during the exfoliation process [79].

### 3.4. Redox Exfoliation

**Figure 3:**
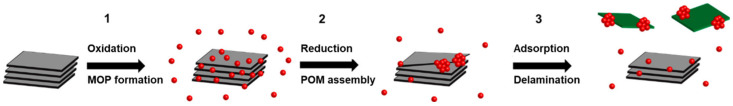
Mechanism of redox exfoliation via POM assembly. Reprinted in part with permission from Ref. [80]. Copyright 2020, American Chemical Society.

Redox exfoliation differs from the aforementioned methods in both mechanism and the properties of the resulting dispersions [80,81]. In this approach, exfoliation is driven by Coulombic repulsion between charged species adsorbed on the surfaces of LTMD flakes. Briefly, redox exfoliation proceeds through a three-step process (Figure 3). Initially, LTMD powders are treated with cumene hydroperoxide (CHP), a mild oxidant, in an anhydrous polar aprotic solvent (acetonitrile), leading to the formation of metal oxide precursors (MOPs). Subsequently, the slurry containing LTMDs and MOPs is treated with hydroquinone (HQ), a mild reducing agent, or NaBH_4_, promoting the conversion of MOPs into polyoxometalates (POMs).

Oxidation occurs predominantly at the edges of LTMD flakes, where POM assembly is initiated. The accumulation of POMs at these sites generates sufficient Coulombic repulsion to facilitate interlayer separation. Simultaneously, the charged POMs act as surfactants, stabilizing the exfoliated flakes in dispersion [82]. Owing to the highly negative charge of POMs, the surfaces of the resulting LTMD flakes are also negatively charged.

Despite the presence of surface charge, redox-derived dispersions exhibit characteristics distinct from those obtained via direct reductive intercalation. In the latter case, injected charges originate from modifications to the electronic structure of LTMDs and can be regarded as an *n*-doping effect. In contrast, in redox-derived dispersions, surface charges arise primarily from the adsorption of POMs without direct electron injection into the LTMD lattice, thereby allowing the materials to retain their semiconducting nature. Furthermore, studies have demonstrated that POMs strongly withdraw electrons from LTMDs, thereby inducing a *p*-doping effect in conjunction with a certain degree of tensile strain on the LTMD surface [31,32].

Overall, redox-exfoliated LTMD dispersions are characterized by the preservation of semiconducting behavior, the presence of a negative surface charge imparted by adsorbed redox products, and the formation of defects as an inherent consequence of the redox process. In addition to providing electrostatic stabilization, adsorbed POMs induce tensile strain and a *p*-doping effect in the LTMD lattice [83].

### 3.5. Classification of Exfoliated LTMD Dispersions

**Scheme 1:**
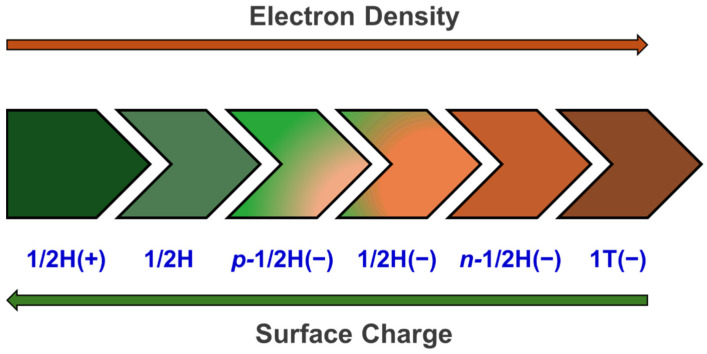
Proposed classification of exfoliated Group VI LTMD dispersions based on crystallographic phase, electron density, and surface charge.

Collectively, exfoliated dispersions of Group VI LTMDs can be systematically classified according to crystallographic phase, electron density, and surface charge, as illustrated in Scheme 1. The crystallographic phase distinguishes between the metallic 1T phase, the semiconducting 1/2H phase, or mixtures of the two. Electron density encompasses undoped 1/2H LTMDs, *p*- or *n*-doped 1/2H LTMDs, as well as intrinsically electron-rich 1T LTMDs. Surface charge arises either intrinsically electron structure of the exfoliated material or extrinsically through the adsorption of surfactants. For example, 1T LTMDs typically exhibit a negative surface charge due to their excess electrons, while *n*-doped 1/2H LTMDs acquire negative surface charge as a direct consequence of electron doping. In contrast, undoped 1/2H LTMDs might also display a negative surface charge when stabilized by adsorbed surfactants; such systems are distinguished from doped counterparts primarily through an assessment of electron density.

Based on these considerations, exfoliated LTMDs obtained via simple surface–solvent matching mechanisms in the absence of surfactants are defined as a reference system. This reference is defined with respect to crystallographic phase (1/2H phase), doping state (undoped), and surface properties (free of surface adsorbates), which are governed solely by interactions with the dispersing solvent. Owing to its minimal chemical and structural complexity, this system is denoted as 1/2H. From this baseline, exfoliated 1/2H LTMD dispersions are further classified as 1/2H(+) or 1/2H(−), depending on whether positively or negatively charged surfactants are present at the surface, without altering the electron density of the material. Additional classification based on electron density yields *p*- or *n*-doped 1/2H LTMDs, designated as *p*-1/2H(−) and *n*-1/2H(−), respectively. Finally, 1T LTMDs, labeled as 1T(−), represent systems with the highest electron density and an intrinsically negative surface charge. A comprehensive overview of this classification scheme is provided in Table 1. Herein, the term “intrinsic” refers to the material in its native state, without any modifications, and typically used in reference to the crystallographic phase, such as 1/2H and 1T LTMDs. However, in this manuscript, “intrinsic” is used specifically to describe a material surface that is free of adsorbates. The original electron density of material (1/2H phase) without modification is instead referred to as “undoped”.

### 3.6. Beyond Exfoliation Techniques: Additional Influencing Factors

#### 3.6.1. Solvents

Dispersing solvents play a critical role in determining the stability and quality of LTMD dispersions. Due to the inherently hydrophobic nature of LTMD surfaces, anhydrous aprotic solvents are generally required to achieve stable colloidal dispersions. In the absence of specific surfactants, LTMD nanoflakes are unstable in water and fail to maintain a colloidal state. Several studies have demonstrated that prolonged exposure to water promotes the formation of MOPs, POMs, or bulk metal oxides [57,91,92,93].

**Figure 4:**
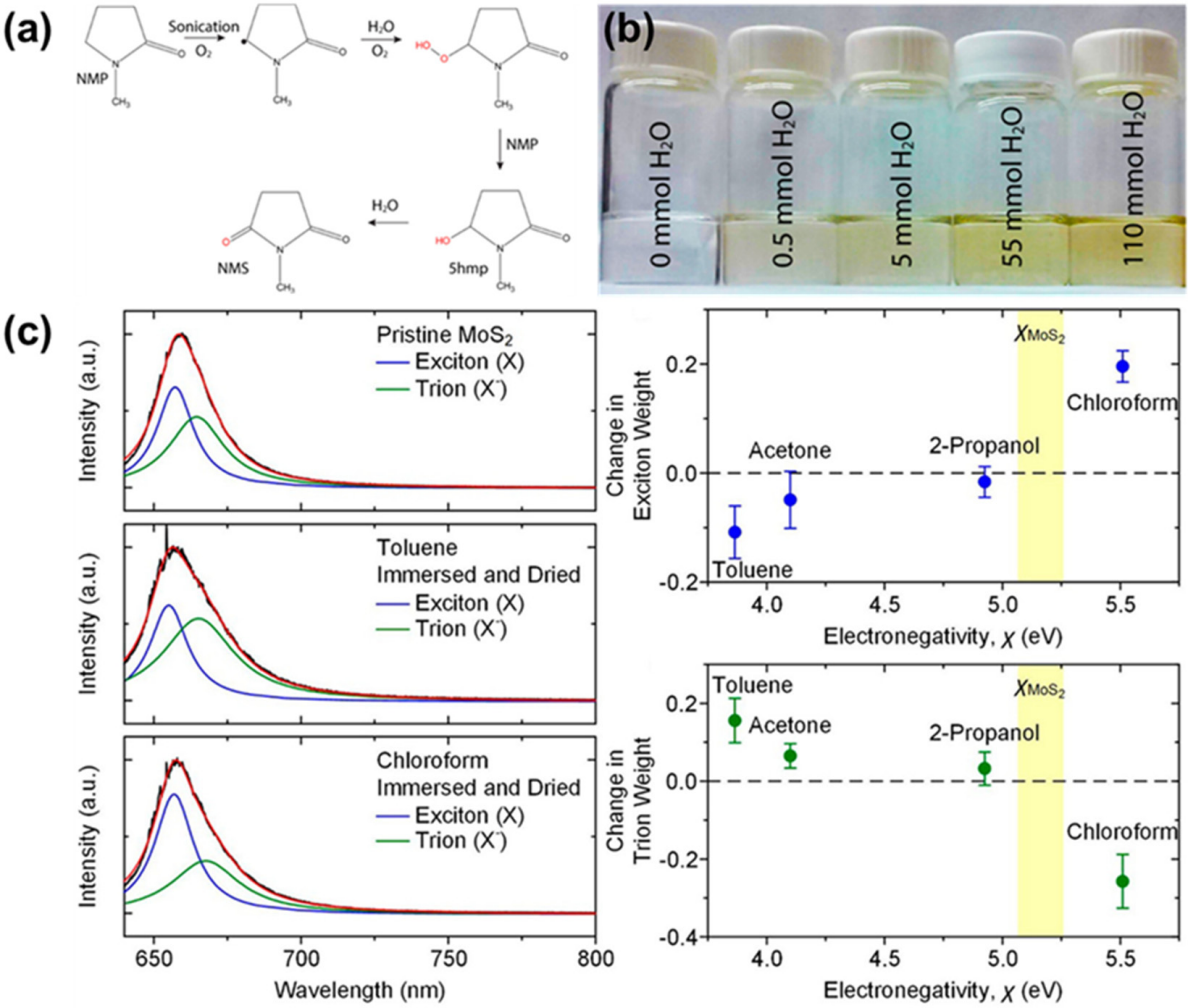
(**a**) Decomposition of NMP in the presence of water, generating a hydroperoxide intermediate; (**b**) concentration of the hydroperoxide with the addition of water. Reprinted in part with permission from Ref. [57]. Copyright 2016, American Chemical Society. (**c**) PL spectra of pristine and solvent-immersed MoS_2_ monolayers, and the corresponding change in exciton and trion weights of MoS_2_ as a function of solvent electronegativity. Reprinted in part with permission from Ref. [79]. Copyright 2016, American Chemical Society.

In 2015, Jawaid and co-workers reported that the introduction of water into anhydrous *N*-methyl-2-pyrrolidone (NMP), a commonly used solvent for LPE, led to the formation of hydroperoxide intermediates that oxidized LTMDs (Figure 4a,b) [57]. This exfoliation pathway was later rationalized as a redox-driven process and subsequently developed into a formal redox exfoliation protocol [80,81]. Notably, in mixed ethanol/water systems, spontaneous auto-oxidation of MoS_2_ was observed as the initial MoS_2_ concentration increased, as indicated by time-dependent color changes in the supernatant, indicative of POM formation [93]. Concurrent pH monitoring of the supernatant further supported the involvement of water in oxidative pathways [93]. Over-oxidation ultimately resulted in the formation of metal oxide products [91].

In exfoliated LTMD dispersions stabilized primarily by Coulombic repulsion—such as redox-derived 1/2H LTMDs or 1T LTMDs—the presence of water can induce desorption of charged surface species or charge depletion through oxidation, leading to colloidal destabilization. In strictly water-free systems, dielectric screening effects become the dominant factor governing the optical and electronic properties of exfoliated LTMDs. These effects are mediated by differences in electronegativity between the dispersing solvents and the LTMDs (Figure 4c) [79].

#### 3.6.2. Starting Materials

**Figure 5:**
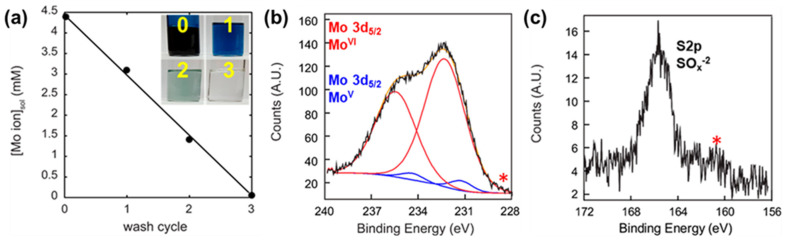
(**a**) Surface impurities of MoS_2_ arise from hydrolysis at surface sites, including edge sites and defects. These impurities are effectively removed by refluxing in an ethanol/acetone mixture. The color of the wash solution varies from blue to green (the numbers indicate washing steps), reflecting the oxidation state of soluble Mo ions. The concentration of metal-ion-based impurities can be quantified using a thiocyanate assay; after the third washing cycle, the impurity concentration becomes negligible and reaches the detection limits of both the instrumentation and the analytical method. (**b**,**c**) X-ray photoelectron spectroscopy (XPS) spectra of the Mo 3d and S 2p regions indicate the presence of Mo^V^ (3d_5/2_ 231 eV), Mo^VI^ (3d_5/2_ 232 eV), and sulfate species (166 eV), consistent with the reported hydrolysis pathway. The asterisks indicate the expected position of Mo^IV^ (3d_5/2_ 228 eV) and sulfide (162 eV) species, which shows that the surface impurities are mainly composed of oxidized sulfur species rather than MoS_2_ entering the liquid phase. Reprinted in part with permission from Ref. [79]. Copyright 2020, American Chemical Society.

**Figure 6:**
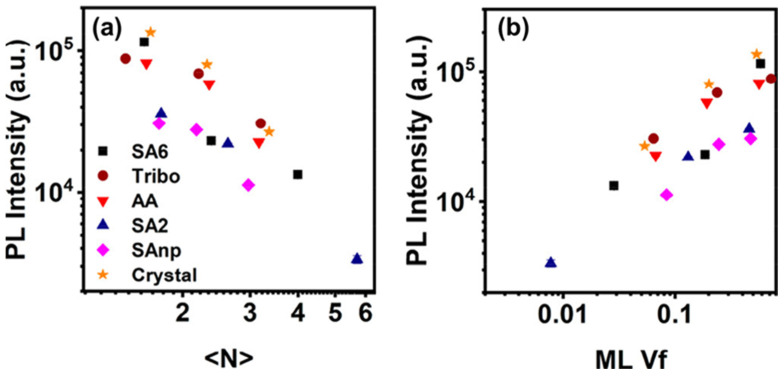
(**a**) Integrated PL intensities of exfoliated MoS_2_ dispersions as a function of the average thickness <N> and (**b**) monolayer volume fraction (λ_excitation_ = 440 nm, 20 °C). PL intensities are calibrated to the MoS_2_ concentration. The MoS_2_ powder sources used for exfoliation, denoted as SA2, SA6, Tribo, AA, SAnp, and Crystal, are listed in Table S1. Reprinted in part with permission from Ref. [94]. Copyright 2019, American Chemical Society.

Surface impurities are present in all Group VI LTMD powders. The majority of these impurities consist of oxidative byproduct formed through hydrolysis of LTMDs at layer edges or in-plane defects, including metal oxides, oxo species, POMs, and MOPs. These impurities have been reported to be effectively removed through multiple washing cycles using an ethanol/acetone mixture, while metal-ion-based impurities can be quantified using the thiocyanate assay (Figure 5). The concentration of impurities increases with increasing surface area, with smaller grain-size powders exhibiting larger surface areas and, consequently, higher impurity concentrations [80]. As a result, surface impurities contribute to variations in the optical properties of LTMD dispersions obtained from different powder sources, as well as differences in exfoliation yield (Figure 6 and Supplementary Materials Table S1) [59,94]. This effect is pronounced across all LPE techniques.

However, solution-based washing processes cannot completely eliminate all surface species. For example, large quantities of MoS_2_ are commonly produced via chemical purification of molybdenite ore as an intermediate step in molybdenum production. After flotation separates metallic minerals, acid leaching yields MoS_2_ powder with a purity exceeding 98.5%, while the remaining insoluble fraction primarily consists of MoO_x_, SiO_2_, Fe, C, and S. These residual impurities have been reported to be effectively removed by conversion into gaseous species through reductive thermal treatment, as recently proposed [95].

Figure 6 illustrates the work of Backes and co-workers, who investigated the impact of starting material quality on the optoelectronic properties of LPE MoS_2_ dispersions. For each dispersion, a centrifuge cascade technique was employed to obtain three fractions collected at screening centrifugation rates of 2000–5000, 5000–10,000, and 10,000–30,000 g. The fraction collected at 10,000–30,000 g was enriched in monolayers. As shown in Figure 6a, the PL intensity was highest for the monolayer-rich fraction; however, substantial variations were observed between dispersions derived from different bulk MoS_2_ powder sources. This observation was further examined by normalizing the PL intensity to the monolayer volume fraction in each dispersion (Figure 6b). The authors attributed the observed discrepancies to differences in defect densities and impurity levels in the starting bulk powders, which vary between sources. These defects and impurities act as non-radiative recombination centers, resulting in PL quenching in exfoliated MoS_2_ monolayers. The chemical compositions of the MoS_2_ powder sources, determined by energy-dispersive X-ray spectroscopy (EDX), are summarized in Table S1.

#### 3.6.3. Heterogeneous Flake Size and Thickness

**Figure 7:**
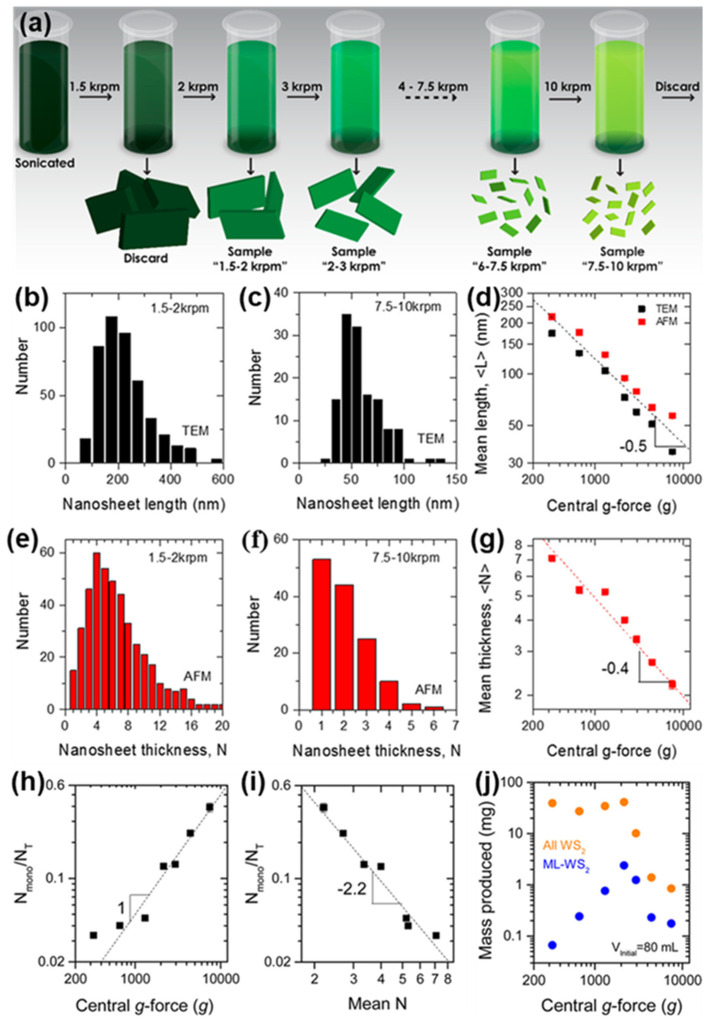
(**a**) Schematic illustration of the centrifugation cascade process. Statistical analysis of the dimensions of exfoliated tungsten disulfide (WS_2_) fractions: flake length and thickness distributions for dispersions obtained at centrifugation rates of (**b**,**e**) 1500–2000 rpm and (**c**,**f**) 7500–10,000 rpm. (**d**) Mean flake length (L) and (**g**) thickness (N) as a function of the applied centrifuge force. Monolayer population data extracted from statistical atomic force microscopy (AFM) analysis over a range of centrifugation conditions. (**h**) Number fraction of monolayers, N_mono_/N_T_, as a function of the central centrifuge force, where N_mono_ is the number of monolayers and N_T_ is the total number of flakes. (**i**) Relationship between N_mono_/N_T_ and the mean nanosheet thickness, N. (**j**) Total mass of WS_2_ and WS_2_ monolayers produced in each fraction as a function of centrifuge force. Reprinted in part with permission from Ref. [88]. Copyright 2016, American Chemical Society.

A broad distribution of flake size and thickness remains a primary challenge for solution-based exfoliation methods. Although intercalation-driven and microwave-assisted exfoliation techniques can partially narrow the thickness and lateral size distributions of 2D flakes, LPE and redox exfoliation approaches are particularly affected by this limitation. To mitigate this issue and obtain monolayer-rich fractions, centrifuge cascade methodologies are commonly employed (Figure 7a) [69,88]. This approach involves systematically adjusting the centrifugation force and duration while selectively collecting the upper supernatant (typically the top two-thirds) after each centrifugation step. Alternatively, multiple centrifugation steps might be applied to achieve more efficient separation. The centrifugation force primarily sorts flakes according to their effective mass, which depends on both the thickness and lateral dimensions, with lateral size being the dominant factor (Figure 7b–g) [88,96]. Consequently, monolayer-rich fractions typically consist of smaller flakes; however, this does not imply that fractions containing larger flakes are devoid of monolayers (Figure 7h–j).

To achieve more precise control over thickness distributions, copolymer-assisted density gradient ultracentrifugation has been employed to separate nanoflakes according to buoyant density [97]. In this method, nanoflakes sediment through a density gradient medium until they reach an isopycnic point at which their buoyant density matches that of the surrounding medium. Although this technique enables efficient separation of flakes with homogeneous layer-by-layer thickness, it is time-intensive and yields relatively small quantities of isolated materials. Moreover, the commonly used gradient medium, iodixanol, has a finite maximum buoyant density, which might limit the applicability of this approach for certain material systems [98]. As a result, size and thickness heterogeneity remains an inherent challenge, as these two parameters are intrinsically correlated [99].

**Figure 8:**
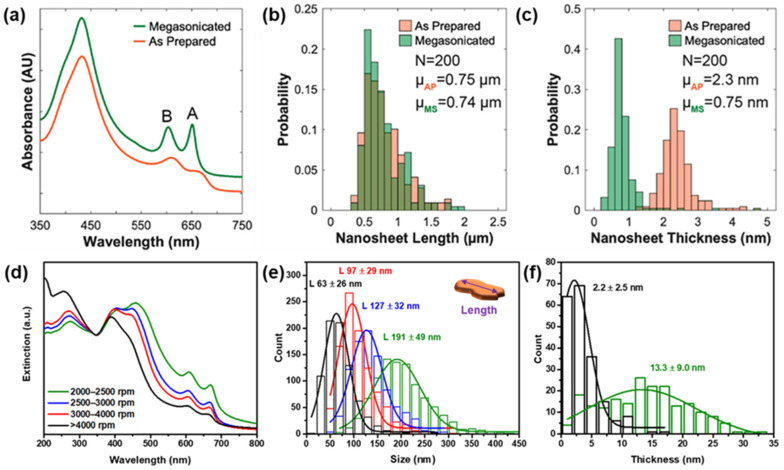
(**a**) Extinction spectra of as-prepared and mega-sonicated MoS_2_ dispersions. (**b**,**c**) Corresponding lateral size and thickness distributions of exfoliated flakes obtained from the as-prepared and mega-sonicated dispersions. Reprinted in part with permission from Ref. [89]. Copyright 2023, American Chemical Society. (**d**) Normalized extinction spectra of four exfoliated MoS_2_ dispersions obtained using different ranges of centrifugation rates. (**e**) Corresponding lateral size distributions of exfoliated flakes from the fractions shown in (**d**). (**f**) Corresponding height distributions of exfoliated flakes from two fractions obtained at 2000–2500 rpm (black) and >4000 rpm (green), as shown in (**d**). The color scheme used in the plots corresponds to that in (**a**) to facilitate visual correlation between data sets. Reprinted in part with permission from Ref. [100]. Copyright 2025, American Chemical Society.

Recently, Hersam and co-workers proposed a post-exfoliation treatment based on mega-sonication to further refine flake thickness distributions [89,90]. The key distinction between mega-sonication and conventional ultrasonication (bath or probe sonication) [88,96,99] lies in the cavitation mechanism governing exfoliation. In both cases, acoustic waves induce pressure fluctuations that lead to bubble formation and dynamics within the liquid medium. While ultrasonication operates at frequencies above 18 kHz and relies on inertial cavitation—where vapor-filled bubbles collapse violently and generate shockwaves that induce delamination and fragmentation—mega-sonication employs much higher frequencies (>350 kHz). Under these conditions, gas-filled bubbles undergo stable cavitation and grow via rectified diffusion, promoting controlled delamination from flake edges. Using this approach, the authors demonstrated that mega-sonication increases the monolayer fraction while largely preserving lateral flake dimensions (Figure 8a–c). This strategy provides an additional route for producing monolayer-rich dispersions while retaining size selectivity through conventional centrifuge cascade processing.

The lateral dimensions of 2D flakes are generally considered to have a negligible influence on the electronic and optical properties of LTMDs compared with the dominant effect of thickness. However, below a critical size threshold, LTMD flakes exhibit molecular-like behavior analogous to that observed in quantum dots [101,102,103,104,105]. In addition, a pronounced blue shift in excitonic transitions is observed in ultraviolet–visible (UV–vis) extinction and PL spectra as the lateral dimensions decrease [73,106]. This effect is further complicated by the fact that smaller flakes are typically enriched in monolayers. Consequently, careful interpretation of excitonic peak positions in UV–vis spectra is required to avoid misattribution of thickness- and size-dependent effects, as discussed in the following section.

Finally, the lateral dimensions govern the surface density of functionalized ligands on LTMD flakes. To maximize surface coverage, ligand packing density varies with flake area and indirectly reflects the surface structure of the surrounding organic environment. These structural variations are further influenced by factors such as vdW dimensions, interchain interactions, and ligand orientation, as discussed in Section 5 [100]. The distributions of flakes size and thickness obtained using different exfoliation techniques are summarized in Table S2.

### 3.7. Characterization: Consideration Beyond Common Interpretation

This section identifies and discusses issues related to data interpretation and sample preparation. These considerations are based on reported values from both earlier and recent studies. Table S3 summarizes the techniques most commonly employed to characterize the properties of exfoliated dispersions of 2D materials. For comprehensive guidance on the characterization of 2D material dispersions, the step-by-step protocols and guidelines developed by the Coleman group are recommended [107,108].

#### 3.7.1. Ultraviolet–Visible Spectroscopy

In 2014, a rapid and accessible methodology based on UV–vis spectroscopy was proposed to determine the lateral dimensions and thickness of exfoliated MoS_2_ and WS_2_ flakes [69]. In this study, centrifuge cascade fractionation was employed to obtain a monolayer-enriched fraction alongside additional fractions with broad thickness distributions. Through extensive statistical analysis of flake thickness, measured by AFM, and lateral dimensions, determined by transmission electron microscopy (TEM), a mathematical model was developed to infer the average thickness and size of a flake population within a given fraction.

Several limitations affecting the validity of these equations can be identified, including: (1) a restricted flake size range (~70–350 nm), (2) a thickness limitation to fewer than ten layers, and (3) the use of centrifugation rates that depend on both size and thickness, implicitly assuming interdependence between lateral dimensions and thickness in dispersions [69,106]. A critical concern relates to the A-excitonic peak position (λ_A_), which is used to estimate the average thickness. This parameter has been shown to vary on a case-by-case basis when the lateral dimensions of monolayer flakes exceed a certain threshold, extending into the micrometer scale [7,77,89,109].

More recently, our investigation into the surface functionalization of exfoliated MoS_2_ and WS_2_ flakes revealed that a subset of LTMD monolayers persisted across multiple cascaded fractions obtained at different centrifugation rates [100]. Notably, the extinction spectra of these fractions, including λ_A_, exhibited a blue shift, as commonly reported, indicating a dominant size effect rather than a dependence on thickness (Figure 8d–f).

A related example is provided by the work from Mark Hersam’s group, who employed mega-sonication. In their study, an as-prepared mono- to bilayer-rich fraction consisted predominantly of flakes with lateral dimensions of approximately 750 nm, which fall outside the previously established model range [69]. This fraction, obtained via electrochemically induced molecular intercalation, exhibited a λ_A_ of 675 nm. Following post-treatment with mega-sonication, which increased the monolayer content, λ_A_ further shifted to 665 nm, while the excitonic transitions in the lower wavelength region (350–550 nm) remained unchanged (Figure 8a) [89]. In addition, the shape of the A-excitonic peak in the mega-sonicated fraction differed markedly from that of the as-prepared fraction. To date, these results represent the first example of a direct comparison between two fractions that differ primarily in thickness while explicitly disregarding the associated trade-off in lateral dimensions. Consequently, both qualitative and quantitative thickness evaluations based on UV–vis spectroscopy should be performed with caution and are best complemented by AFM measurements.

#### 3.7.2. Atomic Force Microscopy

**Figure 9:**
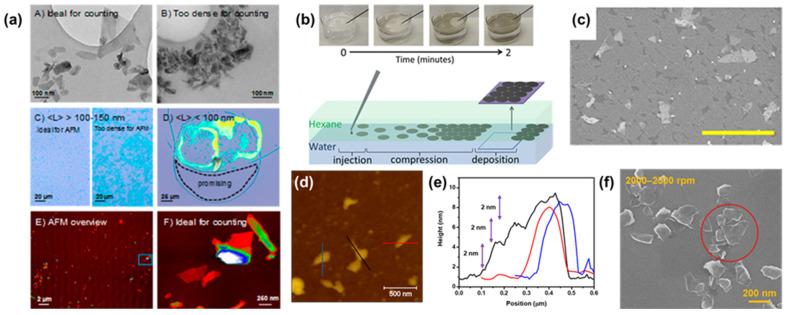
(**a**) TEM images of a MoS_2_ thin film prepared by simple drop-casting, a method prone to flake aggregation (top). Optical microscopy images of aggregated MoS_2_ flakes deposited onto a SiO_2_/Si substrate and captured within an AFM system (middle). Representative AFM measurement illustrating the challenges associated with aggregation during drop-casting (bottom), which restricts analysis to selected regions and significantly increases measurement time. Reprinted in part with permission from Ref. [108]. Copyright 2017, American Chemical Society. (**b**) Schematic illustration of the self-assembly thin-film formation technique employing the interface between two immiscible solvents. (**c**) TEM image of a thin film composed of flat-lying MoS_2_ flakes (scale bar: 5 μm). Reprinted with permission from Ref. [112]. Copyright 2020, American Chemical Society. (**d**,**e**) AFM image of flat-lying MoS_2_ flakes deposited on a SiO_2_/Si substrate and the corresponding height profile of selected nanosheets, revealing a staircase configuration characterized by discrete step heights. (**f**) Scanning electron microscopy (SEM) image of flat-lying MoS_2_ flakes; the edge contrast inherent to SEM imaging highlights incomplete exfoliation and the resulting staircase configuration of few-layer MoS_2_ flakes. Reprinted in part with permission from Ref. [100]. Copyright 2025, American Chemical Society.

The thickness of exfoliated 2D flakes can be directly measured using AFM. However, the sample preparation process, which typically involves drop-casting onto flat substrates such as Si or SiO_2_/Si, often leads to flake aggregation and random orientations rather than a uniform flat-lying configuration (Figure 9a). As a consequence, AFM analysis becomes time consuming, and data interpretation is further complicated by the isotropic distribution of 2D flakes. It has been demonstrated that the orientation of 2D flakes can be confined at the interface between two immiscible solvents, such as hexane and water, using a self-assembly thin-film formation technique (Figure 9b,c). To minimize interfacial tension between the two phases, flakes preferentially adopt a flat-lying alignment, thereby maximizing surface exposure to both media and forming a continuous thin film. Gentle scooping of the film from below enables effective transfer onto various substrates while preserving the flat-lying configuration [110,111,112,113,114]. This approach mitigates issues associated with isotropic flake distribution and establishes a standardized method for reliable thickness measurements and accurate data interpretation.

Beyond flake geometry and substrate arrangement, AFM data interpretation requires additional consideration, as AFM measurements rely on repulsive interactions between the probe tip and the sample surface. In solution-processed dispersions, residual species such as solvent molecules, surfactants, and redox by-products may adsorb onto the flake surface and cannot be completely removed. This can lead to “tip-fooling” effects, in which the AFM tip misidentifies the true LTMD surface due to the presence of physiosorbed species. As a result, the measured thickness of a monolayer often exceeds that of an ideal, pristine monolayer. To address this issue, the use of an internal reference is essential. Incomplete exfoliation of few-layer flakes naturally gives rise to staircase-like configurations (Figure 9d–f), in which the consistent step height between adjacent layers provides a reliable metric for determining monolayer thickness in solution-processed samples [69,80].

#### 3.7.3. Transmission Electron Microscopy (TEM) and Scanning Electron Microscopy (SEM)

TEM and SEM are widely used techniques for investigating material morphology and are commonly employed to determine the lateral dimensions of exfoliated 2D flakes. In addition to size analysis, further information can be extracted based on their distinct imaging principles. TEM images are formed by detecting electrons transmitted through the specimen, whereas SEM images are generated by detecting secondary and backscattered electrons emitted from the sample surface.

Due to the typical geometric orientation of 2D flakes—being standing up or lying flat—statistical analyses of lateral dimensions must be performed with care. This orientation-related variability is a common challenge associated with conventional sample preparation methods. However, when the self-assembly thin-film formation technique is employed, both TEM and SEM can achieve comparable accuracy in lateral size measurements (Figure 9c,f). In SEM imaging, edge contrast is inherently observed due to the interaction of backscattered and secondary electrons with surface features. These edge effects provide direct evidence supporting incomplete exfoliation, which gives rise to the staircase configurations observed in AFM measurements (Figure 9f). In contrast, when samples are predominantly composed of monolayer flakes, TEM is generally preferred over SEM, as the limited backscattered electron signal in SEM can make image focusing and edge definition more challenging.

#### 3.7.4. X-Ray Photoelectron Spectroscopy

**Figure 10:**
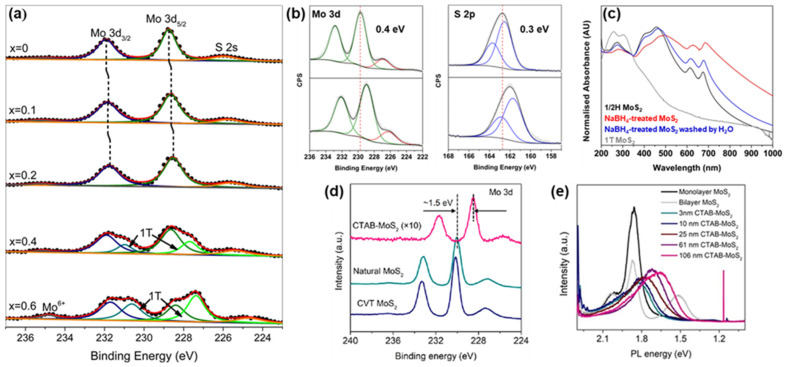
(**a**) XPS spectra of Li-intercalated MoS_2_, showing a shift in the Mo 3d doublet peaks toward lower binding energies during the phase transition from the 1T phase to the 1/2H phase. Reprinted in part with permission from Ref. [22]. Copyright 2016, American Chemical Society. (**b**) XPS spectra of pristine 1/2H MoS_2_ and 1/2H MoS_2_ treated with NaBH_4_. The Mo 3d and S 2p doublet peaks shift toward lower binding energies by approximately 0.4 eV and 0.3 eV, respectively. However, NaBH_4_-treated MoS_2_ retains semiconducting behavior, as evidenced by the presence of the A-excitonic transition in the UV–vis extinction spectra shown in (**c**). Reprinted in part with permission from Ref. [23]. Copyright 2024, Wiley-VCH GmbH. (**d**) XPS spectra of chemical vapor-deposited (CVD) 1/2H MoS_2_ (CVT MoS_2_), pristine 1/2H MoS_2_, and CTAB-intercalated MoS_2_ obtained via electrochemical intercalation. The Mo 3d doublet peaks in the intercalated structure exhibit a pronounced shift toward lower binding energies of approximately 1.5 eV. (**e**) The intercalated structures display PL emission, a characteristic signature of semiconducting MoS_2_ monolayers. Reprinted in part with permission from Ref. [87]. Copyright 2023, American Chemical Society.

The oxidation states of constituent elements are commonly inferred using X-ray photoelectron spectroscopy (XPS). Based on binding energy positions and spectral line shapes, tentative assessment of the structural phase of Group VI LTMDs, such as the 1T or 1/2H phase, can be made. For example, in MoS_2_, the Mo 3d_5/2_ and 3d_3/2_ core-level peaks shift by approximately 0.8 eV toward lower binding energies when transitioning from the 1/2H phase to the 1T phase (Figure 10a). This shift is indicative of increased electron density and is consistent with the metallic character of the 1T phase, in contrast to the semiconducting nature of the 1/2H phase. Additionally, distortions in spectral line shapes may suggest the presence of biphasic mixtures [22]. However, recent studies have indicated that such interpretations are not universally applicable, and caution should be exercised when assigning phases based solely on XPS analysis [23,87].

Krajewska and co-workers reported that the reduction of MoS_2_ using a mild reductant, such as NaBH_4_ in an aqueous medium, yielded semiconducting MoS_2_ flakes accompanied by a binding energy shift of approximately 0.3 eV (Figure 10b,c) [23]. The resulting exfoliated MoS_2_ flakes were partially reduced, leading to defect formation, increased electron density, and a negatively charged surface, while retaining the intrinsic semiconducting phase. Overall, the XPS spectra exhibited a uniform shift relative to the pristine material, without observable deformation of the spectral line shape. This behavior is indicative of an *n*-doping effect rather than a phase transition. This observation aligns with another reported study in which a substantial larger shift toward lower binding energy (~1.5 eV) was observed following electrochemical intercalation, reflecting more extensive electron injection (Figure 10d) [87]. Notably, PL emission was detected from the intercalated materials, signifying semiconducting, monolayer-like behavior (Figure 10e).

#### 3.7.5. Raman Spectroscopy

Thickness-dependent Raman features are among the characteristic signatures of LTMDs at the monolayer architecture. In general, LTMDs exhibit two intrinsic vibrational modes: the in-plane E^1^_2g_ mode and the out-of-plane A_1g_ mode. The number of layers in an LTMD flake can be estimated from the separation between these two peaks (Table 2) [115,116,117,118,119]. It should be noted that most reported thickness determination approaches are based on large-area LTMD sheets with lateral dimensions in the micrometer range. In the case of dispersions exfoliated materials must be deposited as thin films prior to measurement, which introduces additional complexity in data interpretation. Restacking of flakes during film formation and adsorption of residual species can induce peak shifts arising from doping effects, lattice disorder, or surface strain [31,32]. As a result, it becomes challenging to extract reliable thickness information from individual flakes in dispersion. Furthermore, quantitative and qualitative analyses of doping concentrations and strains derived from Raman spectra generally require additional modeling and assumptions. These limitations will be discussed in greater detail in Section 5.

#### 3.7.6. Zeta Potential

The zeta potential of exfoliated LTMD flakes, which reflects their surface charge characteristics, depends on both the intrinsic properties of the LTMDs (e.g., the 1T metallic phase) and the presence of physisorbed surface species typically associated with the 1/2H semiconducting phase. Notably, even in the absence of adsorbed surfactants, LTMD flakes might exhibit a negative surface charge due to residual impurities originating from the precursor LTMD powders. This surface charge has been reported to fall in the range of approximately −5 to −15 mV [81,100].

Upon the introduction of surfactants such as CTAB and sodium dodecyl sulfate (SDS), their headgroup charge plays a dominant role in determining the surface charge of LTMD flakes [68]. CTAB, a cationic surfactant, imparts a positive zeta potential of approximately +57 mV, whereas SDS, an anionic surfactant, yields an opposite zeta potential of approximately −57 mV. In redox exfoliation systems, a negative surface charge induced by the adsorption of POMs has also been reported [80,81]. However, the zeta potential has been shown to vary as a function of flake size and, in certain cases, is influenced by reaction by-products generated during the exfoliation process. This behavior is attributed to differences in stabilization mechanisms that depend on flake dimensions [100]. Larger flakes require higher surfactant coverage to counteract interlayer attractions and maintain colloidal stability, thereby preventing restacking. In contrast, smaller flakes can be effectively stabilized through solvent–surface matching mechanisms and typically require lower concentrations of stabilizers to remain dispersed. These stabilizers can significantly influence subsequent surface functionalization, affecting parameters such as surface organic density and the final structure of the organic layers [100,121]. Nevertheless, the amount of stabilizer present can only be inferred indirectly from zeta potential measurements, and accurate quantification generally requires advanced analytical techniques. A similar trend has been observed for sodium cholate (SC), where the surface charge of LTMD flakes varied with the density of adsorbed surfactant molecules [64].

**Figure 11:**
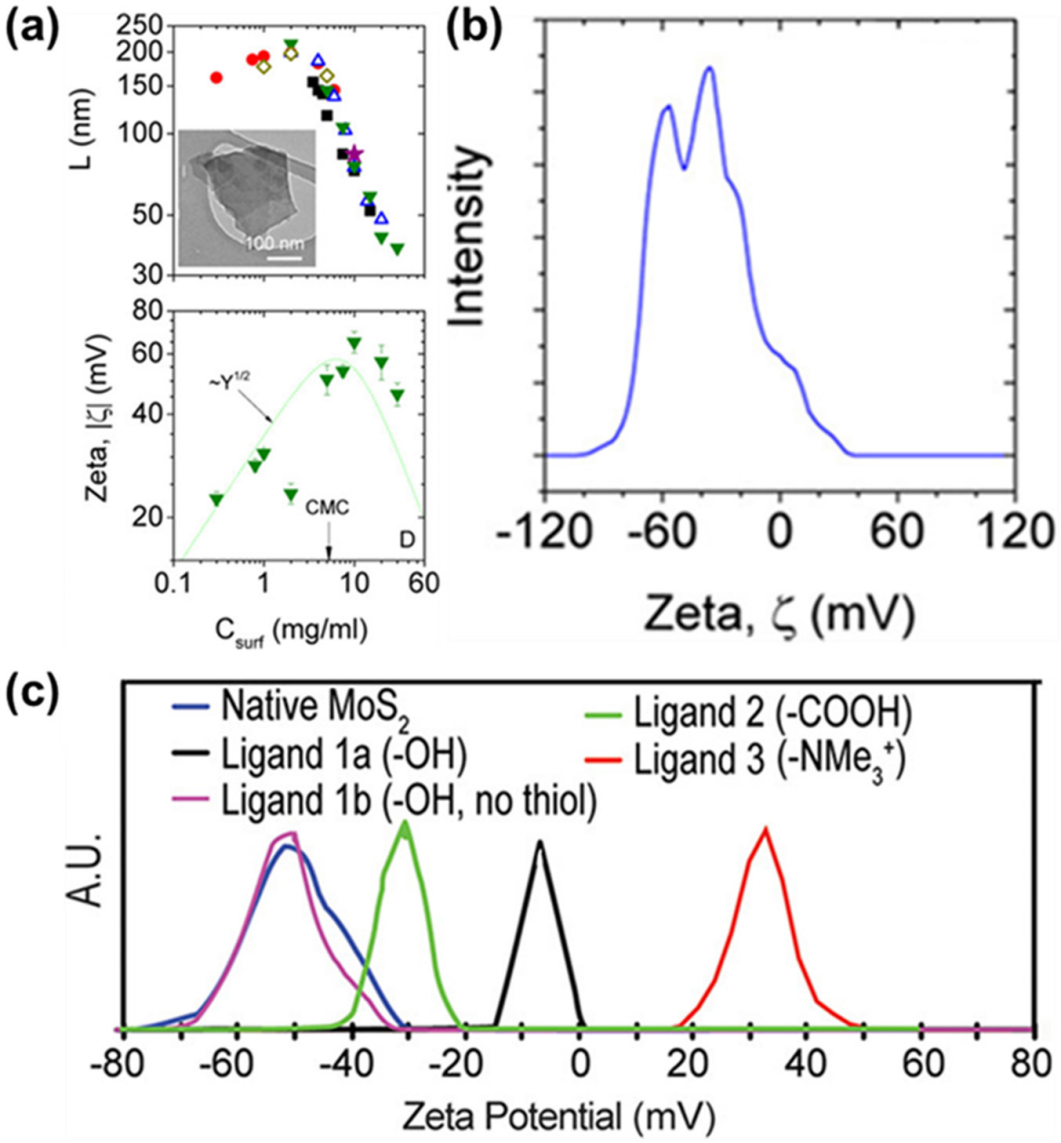
(**a**) Lateral length of exfoliated 1/2H MoS_2_ flakes (**top**) and their corresponding zeta potentials (bottom) as a function of sodium cholate (SC) concentration. The triangle symbols represent measurements obtained at a fixed initial MoS_2_ concentration of 30 mg mL^−1^, shown alongside data acquired at other concentrations. Increasing SC concentration correlates with simultaneous increases in both flake length and zeta potential, suggesting a higher surface coverage of adsorbed SC. However, beyond a threshold concentration near the critical micelle concentration (CMC), both exfoliation yield and flake size decrease, likely due to charge screening effects and micelle formation. (**b**) Broad zeta potential distribution of exfoliated 1/2H MoS_2_ dispersions prepared by liquid-phase exfoliation (LPE) using SC as the surfactant. Reprinted in part with permission from Ref. [64]. Copyright 2015, American Chemical Society. (**c**) Zeta potentials of 1T MoS_2_ and its functionalized derivatives bearing molecules with different tail groups; pristine 1T MoS_2_ is denoted as native MoS_2_. Reprinted in part with permission from Ref. [122]. Copyright 2013, American Chemical Society.

As discussed above, due to the polydispersity in flake size, 1/2H LTMD flakes stabilized by surfactants or redox-active species (e.g., POMs) typically exhibit a broad distribution of zeta potential values (Figure 11a,b). In contrast, 1T LTMD dispersions often display a monomodal or narrower zeta potential distribution centered around approximately −50 mV (Figure 11c) [122]. This distinction highlights fundamental differences in the mechanisms of surface charge generation between 1T and 1/2H Group VI LTMD dispersions.

#### 3.7.7. Experimental Guidelines for the Classification of Exfoliated LMTD Dispersions

Aforementioned challenges in characterization demonstrates that establishing comprehensive experimental criteria for assigning a dispersion to a specific category might be challenging. The challenge arises from the fact that beyond crystallographic phase, electron density, and surface charge, the heterogeneous nature of dispersion—such as variations in thickness, flake size, and the quality of LTMD powders—can also influence the outcome of several characterization techniques, including zeta potential, Raman vibrational modes, and XPS spectral profiles. Additionally, in cases involving physisorbed surfactants on LTMD flakes—classified as 1/2H(+) and 1/2H(−)—the electronic alterations are minimal, leading to insignificant experimental observations in UV–vis absorption, XPS spectral profiles and Raman vibrational characteristics, with only the surface charge being measurably affected. Such subtle electronic alterations are detectable only when monitoring the voltage-current characteristics of a field effect transistor (FET) device fabricated from a large-area LTMD monolayer at micrometer scale. This approach is unattainable to LTMD dispersions and does not fully demonstrate the characteristics of dispersion system.

Considering these limitations and setting aside factors discussed in Section 3.6, we focus primarily on the crystallographic structure, electron density, and surface charge of exfoliated dispersions and tentatively propose the following step-by-step experimental guidelines for assigning dispersions to specific categories:

(1) Internal reference based on 1/2H LTMDs should always be included for comparison.
(2) UV–vis spectroscopy provides partial information on whether dispersions consist of the 1/2H phase, the 1T phase, or a mixture of both. When only the 1T phase is present, excitonic transitions are absent in UV–vis spectra. In contrast, the presence of the 1/2H phase is present—either in a mixture or as the sole phase, results in observable excitonic peaks.
(3) When excitonic transitions are observed in UV–vis spectra, Raman spectroscopy is required to verify the presence of the 1T phase, since its distinctive vibrational modes are characteristic of this phase and absent in the 1/2H phase (detail in Section 5).
(4) When Raman spectroscopy confirms that only 1/2H phase is present, the electron density of material can be evaluated through shifts in binding energy observed by XPS, particularly in the case of *n*-doping (*n*-1/2H(−)).
(5) When XPS spectral profiles display insignificant shifts in binding energy, three possibilities might arise: *p*-1/2H(−), 1/2H(−), or 1/2H(+). However, it should be noted that, to date, only redox-exfoliated LTMDs have been reported to exhibit *p*-doping, typically accompanied by a high content of metal-oxo species compared to that of the 1/2H reference. Finally, 1/2H(−) and 1/2H(+) can be distinguished by zeta potential measurement to evaluate the surface charge.

**Scheme 2:**
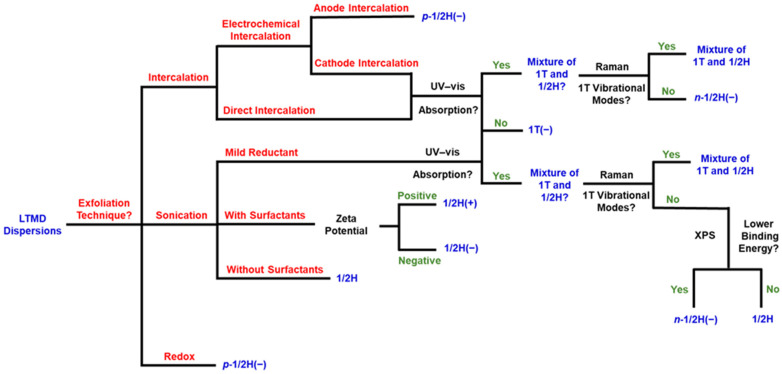
Guidelines to the Classification of Exfoliated LTMD Dispersions.

In addition to experimental characterization, dispersions can also be preliminary categorized based on the exfoliation techniques, as shown in Scheme 2.

## 4. Interplay Between Exfoliation and Functionalization Strategies

To date, efforts toward establishing surface–ligand configurations in Group VI LTMDs can be classified into four main strategies: chemical passivation of chalcogen defects [123,124,125], edge-site covalent linkage [126], physisorption [127,128], and basal-plane surface functionalization [126,129,130,131,132]. The selection of an appropriate functionalization strategy is dictated by the physicochemical properties of exfoliated LTMD flakes, as discussed in Section 3. Both defect passivation and edge-site covalent linkage involve the formation of new bonds at unsaturated sites, the availability of which is regulated by the defect density, whether intrinsically present or generated during exfoliation. In contrast, physisorption predominantly affords surface coverage through weak, non-covalent interactions without the formation of strong chemical bonds. Basal-plane surface functionalization, which involves direct covalent bonding to the crystallographic basal plane of semiconducting Group VI LTMDs, has been rarely reported due to the inherent chemical inertness of the basal surface. Such functionalization typically requires a pre-activation step, which might compromise the crystallographic integrity of LTMDs.

Recent findings from multiple case studies suggest that surface functionalization may encompass a broader range of interactions beyond conventionally defined covalent bonding [43,55,87,121]. These studies indicate that an ionic model can effectively describe Group VI LTMD–ligand interactions, in which the proposed ionic bonding exhibits considerable stability in suitable solvent environments [100]. In aprotic solvents, these ligands strongly adhere to the LTMD surface and can function as dielectric layers that mitigate interlayer coupling, thereby preserving the intrinsic semiconducting properties of LTMD monolayers.

**Scheme 3:**
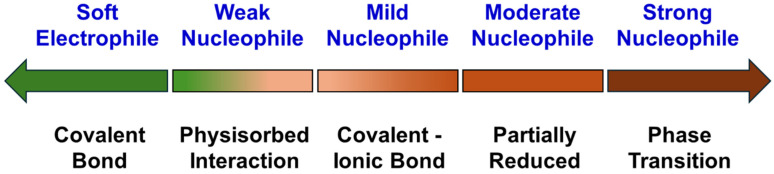
Schematic illustration of Group VI LTMD–ligand interactions as a function of the reducing strength of organic ligands. Reprinted in part with permission from Ref. [100]. Copyright 2025, American Chemical Society.

In this section, we discuss the interactions governing hybridized LTMD–ligand configurations. Building upon previous findings, we tentatively propose that exfoliated dispersions with tunable properties might enable targeted functionalization strategies. Finally, we present new insights into the interaction mechanisms governing LTMD–ligand composites, conceptualized in terms of electrophile–nucleophile interactions, or more broadly, Lewis acid–base chemistry (Scheme 3).

### 4.1. Comparison of LTMD–Ligand Interactions with Electrophile–Nucleophile Interactions

A review of experimental and theoretical studies, spanning early efforts in the chemical exfoliation of Group VI LTMDs to recent advances in exfoliation techniques and functionalization strategies, reveals several key observations:

(1) The phase transformation from the 1/2H phase to the 1T phase is reversible. This transition is primarily driven by crystallographic rearrangement, with electron concentration serving as the dominant factor governing the phase change. The transformation, often induced by strong reducing agents, bears resemblance to a Lewis acid–base process analogous to surface hybridization between electron-donating ligands (e.g., amines and phosphines) and Group V LTMDs [121].
(2) Although the 1T phase of Group VI LTMDs exhibit metallic behavior, it differs from the intrinsically metallic Group IV and V LTMDs in several aspects. For example, Group IV and V LTMDs readily accept electrons from organic ligands such as amines and phosphines [121]. This phenomena has been observed in systems where electron-donating molecules intercalate into single-crystal Group IV and V LTMDs [133]. In contrast, analogous intercalation has not been reported for the 1/2H phase of Group VI LTMDs. Furthermore, 1T Group VI LTMDs tend to donate electrons to electron-withdrawing species (e.g., organohalides), rather than acting as electron acceptors [10,129]. Despite their electron-rich character, 1T LTMDs exhibit strongly negative surface charge, whereas Group IV and V LTMDs typically display near-neutral surface charge. This distinction is likely related to the metastable nature of the 1T phase in Group VI LTMDs, where excessive electron injection might ultimately lead to chemical degradation [50].
(3) While strong reducing agents promote phase transformation, milder reducing agents might facilitate partial electron transfer that enables the formation of robust covalent bonds [134,135,136]. This possibility raises two key questions: (1) If such a mechanism exists, there will may be a defined range of reducing strength in which certain ligands promote bond formation, whereas outside this range—at either higher or lower reducing power—only phase transformation or physisorption might occur; and (2) If covalent bond formation takes place, would Group VI LTMDs retain their semiconducting character or undergo a transition toward metallic behavior?
(4) From an alternative perspective, an electrophilic functionalization pathway has recently been proposed, based on the electron-rich basal plane of semiconducting LTMD flakes. However, this approach requires the use of soft electrophiles, such as maleimide rings containing conjugated C=C bonds [130].

Collectively, these observations raise the following questions:

(1) Can the interactions between Group VI LTMDs and organic ligands be generally described within the framework of electrophile–nucleophile chemistry, and in certain cases, as Lewis acid–base interactions?
(2) If so, what roles do Group VI LTMDs play in these reactions, namely acting as nucleophiles or electrophiles and, correspondingly, as Lewis bases or Lewis acids?

### 4.2. Interactions Between Group VI LTMDs and Nucleophiles

Most reported studies on Group VI LTMDs focus on their interactions with reducing agents. The reducing strength of nucleophiles strongly influences the nature of these interactions, thereby governing the choice of suitable organic ligands and defining the roles of both LTMDs and molecular species in the reaction mechanisms.

#### 4.2.1. Physisorption

The simplest interaction involves physisorption of ligands onto the surface of exfoliated 1/2H Group VI LTMD dispersions. In this context, surfactants (e.g., CTAB, SDS, SC) adsorb onto the basal planes of LTMD flakes and form micellar structures [68]. These adsorbates act as interfacial stabilizers by compensating for the interfacial energy mismatch between the 2D flakes and the dispersing solvent, thereby improving colloidal stability.

Alternatively, several studies have explored the use of thiol-based molecules through coupling chemistry. It has been proposed that chalcogen–chalcogen bridges might form between –SH or –S–S– functional groups and the chalcogenide layers of LTMDs. Although covalent bonding might occur at intrinsic vacancies, edge sites, or other defects [123,124,125,126], chalcogen–chalcogen interactions are generally weak and often yield physiosorbed dithiol molecules [137,138]. Moreover, this approach is limited by the requirement for aqueous or protic solvents and often requires empirical optimization of ligand-to-LTMD ratios to achieve stable dispersions [139,140]. Overall, the effectiveness of thiol-based functionalization is strongly dependent on defect density, which governs the achievable ligand surface coverage [141]. Since defects, vacancies, and edge sites are inherent to exfoliated LTMD dispersions, their density can be further increased through chemical exfoliation to obtain the 1T phase, in which aggressive reduction generates a higher density of structural defects [122,140,142,143].

#### 4.2.2. Covalent Bonding

Whereas thiol-based ligands behave as weak nucleophiles and form relatively labile surface interactions, strong nucleophiles often induce phase transformation, leading to the formation of 1T LTMDs. Consequently, direct bonding between strong nucleophiles and LTMDs is typically not observed. However, 1T LTMDs exhibit pronounced nucleophilic character and readily donate electrons to electrophilic reagents such as epoxide rings [144], organohalides [10,129,132], and radical-based coupling partners including diazonium salts [131,145,146,147,148], and alkyl azide [149], forming robust covalent bonds. This phase transformation, often described as a pre-activation step, enables a high degree of ligand functionalization across the basal planes of LTMDs. In addition, the electronic and surface properties of LTMD flakes can be tuned by varying the ligand tail groups [122].

Once covalent functionalization is established, the 1T phase is stabilized and does not revert to the 1/2H phase [150]. Notably, Yan and co-workers demonstrated that molecular coverage density can be systematically tuned by increasing electron injection into the LTMD lattice. This tunability was achieved by treating LTMDs with a series of metallocene compounds with different reducing strengths, referenced to the Fermi level of LTMDs [151]. In contrast, 1/2H LTMDs generally do not react with these organic electrophiles unless catalyzed by Pd(0) [132]. These findings suggest that elevated electron density and a strongly negative surface charge are critical factors controlling reactivity and molecular coverage density on LTMD surfaces [152]. Therefore, partially reduced semiconducting LTMDs (*n*-1/2H(−)) might also exhibit reactivity under similar conditions [23].

#### 4.2.3. Ionic Bonding

Recent theoretical studies have suggested that neutral carbenes potentially form covalent bonds with LTMD surfaces through orbital hybridization [134,135,136]. Experimental validation has been reported; however, different studies proposed distinct binding sites, suggesting either coordination to chalcogen atoms [153] or binding to transition-metal defect sites [154]. Nevertheless, these findings are significant, as they suggest that robust surface bonding might provide a route for tailoring the properties of Group VI LTMDs. Since carbenes are mild electron donors, these results further suggest that other nucleophilic ligands, such as Grignard reagents (R–MgBr) and organozinc compounds, might also be capable of functionalizing LTMD surfaces. Overall, these observations imply the existence of an optimal range of nucleophile reducing strength, within which strong ligand–LTMD interactions can be achieved. Such interactions might extend beyond physisorption and electron transfer during pre-activation and might involve covalent or ionic bonding.

Several studies have demonstrated the formation of ionic interactions. In 2018, Duan and co-workers revisited superlattice architectures based on diverse 2D frameworks by employing electrochemical intercalation of quaternary alkylammonium cations ([R_4_N]^+^) into the interlayer galleries of single-crystal MoS_2_ and WSe_2_ [55]. Upon applying sufficiently negative potentials to LTMD electrodes, interlayer vdW attractions were overcome, enabling intercalation of [R_4_N]^+^ species and the subsequent formation of superlattice structures. These superlattices exhibited expanded interlayer spacing, as shown by a shift in the diffraction peak toward lower angles relative to the intrinsic (002) reflection corresponding to the native LTMD interlayer distance. This process represents a direct electron-injection pathway, in which the degree of doping is modulated by the steric hindrance of the intercalated cations in accordance with charge-balance mechanism [55,155]. For instance, intercalation of CTA^+^ introduced a higher electron dose and triggered formation of the 1T phase, whereas bulkier cations such as THA^+^ resulted in lower electron injection, thereby preserving the 1H phase and its semiconducting properties. Importantly, the intercalated organic layers effectively passivated interlayer interactions, imparting bulk materials with properties resembling those of isolated semiconducting monolayers.

The ionic character of these hybrid systems was further supported by electrochemical measurements, which showed that organic cation intercalation occurred at lower applied potentials for the electron-rich 1T phase compared with the 2H phase [85]. Notably, although doped electrons could be removed by mild oxidative treatment, the superlattice structures remained intact and preserved semiconducting characteristics [86]. Despite the ability of ionic bonding to modulate LTMD electronic properties, these interactions, which are primarily governed by Coulombic attraction, are intrinsically weak. As a result, the associated organic ligands are prone to detachment upon exposure to protic solvents [54,56,156,157]

**Figure 12:**
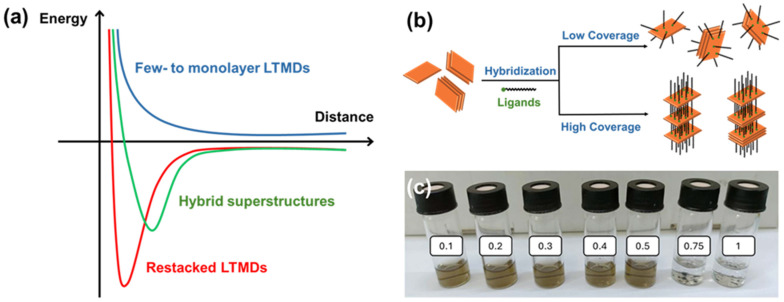
(**a**) Energy scheme of LTMD flakes in dispersions and in restacked structures. (**b**) Colloidal destabilization-induced restacking as a function of surface coverage density. (**c**) Photograph of 1T MoS_2_ suspensions after addition of 0.1, 0.2, 0.3, 0.4, 0.5, 0.75, and 1 equivalent of cholamine chloride (ChNH_2_). At high concentrations, dense adsorption of ChNH_2_ on 1T MoS_2_ flakes disrupts colloidal stability by altering the stabilization mechanism, thereby inducing restacking and formation of an intercalated superlattice structure. Reprinted in part with permission from Ref. [158]. Copyright 2025, Royal Society of Chemistry.

Using a solution-processing approach, our group recently demonstrated that exfoliated 1/2H Group VI LTMDs readily react with masked *N*-heterocyclic carbenes (NHCs), which act as mild nucleophiles [100]. This interaction introduces a small fraction of electrons into exfoliated 1/2H MoS_2_ and WS_2_ flakes, giving rise to *n*-doping without inducing a phase transition. The excess negative charge is compensated by the organic cations of the imidazolium salts, which destabilize the colloidal system and generate an additional potential well that promotes the formation of restacked superlattice structures (Figure 12a). This observation is consistent with previous reports on intercalated systems [158], indicating that organic ligands modify dispersion stabilization mechanisms and promote restacking and aggregation (Figure 12b,c). In aprotic solvents, however, the ligands bind strongly to LTMD surfaces and remain attached even under substantial perturbations such as sonication. These findings enable the formulation of LTMD-based inks and the fabrication of hybrid LTMD–ligand thin films, in which the superlattice architecture and semiconducting monolayer-like properties are preserved [100].

### 4.3. Interactions Between Group VI LTMDs and Electrophiles

To date, only a limited number of studies have reported the successful functionalization of semiconducting Group VI LTMDs using electrophilic molecules. The primary challenge lies in the chemical inertness of 1/2H Group VI LTMDs, which is largely attributed to their fully occupied valence bands. Although the basal planes of these 2D materials are electron-rich, electrophilic attack was rarely observed until a pivotal study in 2019 [130,159,160]. In that study, Pérez and co-workers introduced maleimides, a class of soft electrophiles characterized by an electron-deficient double bond, which are capable of accepting electrons via thiol–ene click chemistry [161]. Their findings provided a new strategy for covalent surface functionalization of exfoliated 1/2H Group VI LTMDs, using MoS_2_ and WS_2_ as representative systems.

Despite the promise of this approach, several challenges remain, particularly regarding the quantification of covalent bond formation. Surface analyses revealed that ligand surface coverage was lower than initially expected. Subsequent investigations showed that the presence of a base, triethylamine (Et_3_N), promoted the formation of a polymeric maleimide adlayer, which originated from the initially covalent attachment of maleimide molecules [162].

It is notable that other electrophiles, such as organohalide derivatives, do not exhibit comparable reactivity. Although halides are effective leaving groups that render electrophilic centers susceptible to nucleophilic substitution, reactions between organohalides and 1/2H LTMDs proceed only in the presence of catalysts [132]. This raises the question of whether electron withdrawal from the LTMD surface destabilizes the system, thereby rendering the reaction thermodynamically unfavorable.

## 5. Characterization Challenges of Group VI LTMD–Ligand Hybrids

Beyond conceptual understanding and experimental demonstrations, characterization remains a major challenge in elucidating LTMD–ligand hybrid systems. The interpretation of characterization data might vary depending on the specific materials under investigation. Furthermore, factors such as sample preparation, material heterogeneity, and experimental conditions can strongly influence the resulting spectra and their interpretation. Therefore, a rigorous understanding of characterization methods commonly applied to functionalized 2D materials is essential. A comprehensive overview of techniques for covalent functionalization has been provided elsewhere [163]. Accordingly, this section focuses on selected case studies that highlight additional analytical challenges and discusses strategies to address them.

### 5.1. Surface Functionalization of Mechanically Exfoliated Group VI LTMDs and Their Dispersions: Key Distinctions

Before addressing the main topic of this section (Section 5.2 and Section 5.3), we briefly outline key distinctions between LTMD-ligand hybrid systems prepared by mechanically exfoliated (ME) LTMDs and those derived from wet-chemistry exfoliated (WE) LTMD dispersions. It is important to note that certain interpretations and characterization observations reported in ME LTMD-ligand hybrids might not be directly transferable to WE LTMD-ligand systems. These differences is critical for accurate data analysis and for guiding the interpretation of WE LTMD-ligand systems, particularly when ME LTMD-ligand hybrids are used as reference models in situations where characterization of WE systems remains challenging. The characterization of ME LTMD-ligand hybrids has been long extensively reported and has reached certain agreements within the field. For a comprehensive overview of these systems, we highly recommend that readers consults two reviews articles [41,164].

#### 5.1.1. Crystallographic Phase–Doping Effects—Heterogeneity

Mechanical exfoliation exploits the weak vdW interactions between stacked monolayers of LTMD crystals. These interlayer forces can be effectively overcome by applying adhesive tape to physically peel the material layer by layer. Repeated exfoliation ultimately yields isolated monolayers with dimensions typically at the micrometer scale. In most studies, the resulting monolayers employed is the semiconducting 1H phase. Since the exfoliation process is purely physical, minimal changes are expected, and the quality of the resulting materials is largely determined by the quality of initial LTMD crystals. Consequently, the ME LTMD monolayers are considered undoped and free from solvent-induced effects, in contrast to WE 1/2H LTMDs. The conversion of ME monolayers from the semiconducting 1H phase to the metallic 1H phase is possible, as reported through treatment with the *n*-BuLi solution [129].

In contrast, rather than conducting experiments on a single monolayer, wet-chemistry exfoliation produces dispersions of exfoliated flakes that exhibit heterogeneity in phase composition (mixture of the 1T and 1/2H phase), doping characteristics (*n*- or *p*-doping), and surface properties. These surface properties are either influenced by solvents or surfactants employed. Additionally, the heterogeneity also manifests in variations in thickness and flake size, which directly dictates the electronic and surface properties of individual nanoflakes within the dispersion. The experimental observation of WE LTMD-ligand hybrids reflect the collective behavior of nanoflakes in dispersion rather than the properties of ME LTMD-ligand hybrids of isolated single layers. As a result, in WE LTMD-ligand systems, it is difficult to attribute observed effects to isolated factors; instead, they should be considered as arising from multiple interdependent factors.

#### 5.1.2. Structures of the Functionalized Hybrids

The fundamental chemistry driving functionalization between ME LTMDs and WE LTMDs might be considered broadly similar. The primary distinction lies in the surface properties and the mechanisms of colloidal stabilization, which is relevant primarily to WE systems. In most cases, functionalization is carried out in the liquid phase. ME monolayers on substrates are immersed in ligand solutions, whereas WE LTMDs are initially produced as dispersions in liquid media and require only the subsequent addition of ligand solutions. However, functionalization of ME monolayers can also be performed in the gas phase [153].

In the case of ME LTMDs, the reactive area is limited to the side of the monolayer exposed to the liquid medium. Because these systems are not influenced by the stabilization mechanism and typically possess relatively large lateral dimensions (at micrometer scale), ordered self-assembled monolayers (SAMs) are formed and ungoverned by kinetic constraints. The formation of SAMs can occur for both physisorbed and covalently bound ligands, whereas the degree of long-range order depends on the intermolecular interactions between ligands [127,128,153,165].

In the case of WE LTMDs, the reactive surface expands to both sides of the nanoflakes. In a liquid medium, functionalization alters the surface characteristics, which changes the colloidal stabilization mechanism and consequently leads to aggregation and restacking phenomena (Figure 12c). Surface adsorbates actively participate in the process and act as kinetic control factors, often giving rise to disordered molecular arrangements and incomplete surface coverage [100,121]. Variations in flake sizes also play a critical role in determining the molecular ordering on the surface [100]. Ultimately, these factors collectively lead to the formation of intercalated structures, in which their interlayer galleries exhibit diverse molecular environments, thereby giving rise to distinctive electronic properties [100].

#### 5.1.3. Characterization

Aforementioned points have largely described the distinctions between ME LTMD-ligand and WE LTMD-ligand systems. Owing to their well-defined large-area monolayers and long-range ordered of molecules, ME LTMD-ligand systems often serve as reference models for evaluating phenomena such as molecular doping effects, molecular ordering effects, and related interfacial processes. For example, the ordering and orientation of molecules bearing functional groups can generate internal dipolar vectors, which either add constructively or cancel out each other depending on their relative alignment [35,36]. Direct charge transfer between molecules also act as a dominant factors influencing the overall performance of hybrid materials [38,40]. As a result, the molecular effects on LTMD monolayers can be evaluated by detectable changes, such as in XPS spectral profiles and Raman spectra. Based on these observations, valuable information regarding to doping behavior and induced surface strain have been inferred and widely accepted among researchers. In the case of WE LTMD-ligand systems, comparable observations are often difficult to obtain due to pre-doping effects introduced during exfoliation, as well as the disordered arrangements of ligands arising from multiple kinetically controlled factors. Although ME LTMD-ligand systems might serve as reference models to guide interpretation and comparative analysis, the corresponding calculation models requires further refinement, which will be discussed in the following section.

Finally, it is important to note that electronic modulation is directly detected by monitoring the working voltage-current characteristics in field effect transistors (FETs). Such device fabrication for such measurements is typically performed in ME LTMD monolayers due to their micrometer-scale dimensions. Conversely, device fabrication based on WE LTMD nanoflakes require thin films, which introduces additional influencing factors, including interflake connectivity, interflake transport, interflake resistivity, and further complicate the interpretation [166,167,168].

### 5.2. Crystallographic Phase–Doping Effects–Surface Strain

#### 5.2.1. Crystallographic Phase

The crystallographic structures of Group VI LTMDs have been investigated using a variety of techniques. TEM [16,169] and scanning tunneling microscopy (STM) [18] provide direct structural information with atomic resolution. However, the limited lateral dimensions of exfoliated flakes, typically on the micrometer scale, constrain data acquisition and require careful experimental design. In addition, dispersion-related issues, including aggregation and sheet folding, can compromise the reliability of structural analyses.

Although TEM and STM provide high-resolution structural information at the level of individual flake, they are inherently limited in representing the overall population of exfoliated materials. In contrast, spectroscopic techniques such as UV–vis absorption spectroscopy and Raman spectroscopy enable population-wide analysis and provide a more representative assessment of optical and structural properties. However, heterogeneous phase mixtures might introduce ambiguities in spectral interpretation. For example, the 1T phase lacks excitonic transitions that are characteristic of the 1/2H phase in UV–vis spectra (Figure 10c).

**Figure 13:**
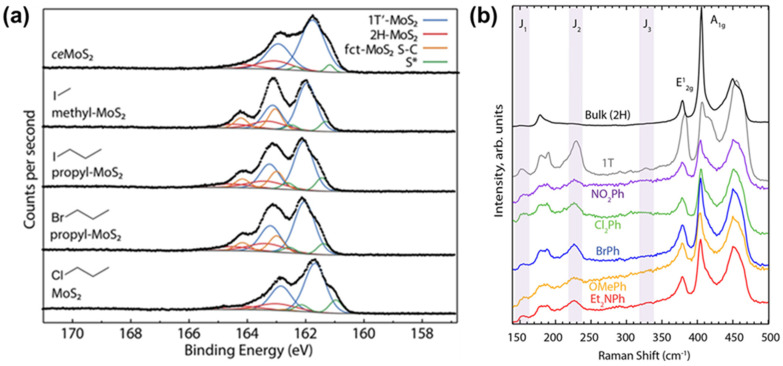
(**a**) XPS S 2p spectra of chemically exfoliated MoS_2_ (via *n*-BuLi) and its functionalized derivatives. Reprinted in part with permission from Ref. [151]. Copyright 2020, American Chemical Society. (**b**) Raman spectra of 2H and 1T MoS_2_, along with functionalized derivatives of 1T MoS_2_. Reprinted in part with permission from Ref. [147]. Copyright 2018, American Chemical Society.

XPS is a valuable technique for assessing phase composition (Figure 10a). However, crystallographic integrity can be modulated by changes in electron density without substantial changes in chemical bonding [23,87]. Raman spectroscopy is particularly effective for identifying the 1T phase due to its distinct vibrational modes [21,170]. As a result, Raman spectroscopy is frequently used for phase identification, particularly given the common coexistence of 1T and 1/2H domains in exfoliated samples (Figure 13). Therefore, for exfoliated Group VI LTMD dispersions and their functionalized hybrids, a combination of UV–vis spectroscopy, Raman spectroscopy, and XPS is recommended to obtain a comprehensive assessment of phase composition.

In Group VI LTMD–ligand hybrids, several important considerations must be taken into account. If covalent bonds are formed, the 1T phase typically remains stable, with no observable phase transition (Figure 13) [129,147,151]. Although the crystallographic structure is preserved, functionalization might redistribute the electron density, leading to the emergence of semiconducting behavior in the hybrid materials [10,129,151]. Similarly, for 1/2H LTMDs, direct covalent bond formation does not appear to induce a phase transition, and no studies to date have reported evidence to the contrary.

In contrast, if the interactions between LTMDs and ligands are purely ionic, semiconducting properties are not expected to emerge when the exfoliated Group VI LTMDs are in the 1T phase [171]. However, if the starting LTMDs retain the 1/2H phase, the resulting hybrids might exhibit semiconducting characteristics even in the presence of substantial *n*-doping (Figure 10d,e) [87]. Under these conditions, shifts in XPS peaks toward lower binding energies (~1.5 eV) should be interpreted with caution and should be corroborated using complementary techniques such as Raman spectroscopy, UV–vis spectroscopy, PL, and related methods.

#### 5.2.2. Doping Effects–Surface Strain

To date, doping effects in Group VI LTMDs and their corresponding hybrids have been most directly evaluated through the fabrication of field-effect transistor (FET) devices. This approach requires large-area LTMD flakes, typically with lateral dimensions of at least 5 μm. Consequently, it poses a limitation for solution-processed LTMD dispersions, which generally yield smaller flakes. Alternatively, indirect evidence of doping can be obtained using XPS and Raman spectroscopy. However, these techniques also face challenges due to the inherent structural and chemical complexity of exfoliated dispersions.

Raman spectroscopy is widely used to monitor shifts in in-plane and out-of-plane vibrational modes, which are characteristic of Group VI LTMDs in both intrinsic and doped states. For example, in MoS_2_ monolayers, the A_1g_ mode is more sensitive to doping, whereas the E^1^_2g_ mode primarily responds to lattice strain [28,29,30]. A downshift of the A_1g_ mode is commonly associated with *n*-doping (phonon softening), whereas an upshift is generally attributed to *p*-doping (phonon hardening) [40]. Similar trends have been reported for other Group VI LTMDs, including WSe_2_ monolayers [35]. Notably, such doping effects can arise even when LTMD–ligand interactions are dominated by weak physisorption and remain reversible [32]. These behaviors have been attributed to multiple mechanisms, including charge transfer and dipole–dipole interactions. However, most studies to date have been conducted on well-defined CVD-grown 1H LTMD monolayers, which are typically regarded as intrinsic and relatively defect-free [41,164]. In contrast, for solution-processed dispersions, simultaneous shifts in both the A_1g_ and E^1^_2g_ modes, together with broadening of their full width at half maximum (FWHM), suggests the coexistence of doping effects and surface- or lattice-induced strain. These phenomena appear to be strongly coupled, making it challenging to decouple and independently quantify contributions from electronic doping and structural perturbation.

##### Exfoliated Group VI LTMD Dispersions

A representative case study examining the vibrational behavior of 1/2H MoS_2_ thin films deposited from different exfoliated dispersions was reported by Busch and co-workers [31]. Their study aimed to investigate variation in the optical properties of deposited thin films, which were strongly dependent on the exfoliation method. In that study, four exfoliated MoS_2_ dispersions were prepared from the same CVT-grown MoS_2_ powder source.

Solvent-mediated exfoliated (SME) MoS_2_ was produced via probe sonication in NMP using a surface/solvent-matching mechanism, yielding 1/2H MoS_2_ [57,58]. Chemically exfoliated (CE) MoS_2_ was obtained through *n*-BuLi treatment, producing 1T MoS_2_ [19]. Subsequent thermal annealing reconverted the material to the 1/2H phase, yielding chemically exfoliated phase-reconverted (CEPR) MoS_2_. Redox-exfoliated (RE) MoS_2_ was prepared through conventional redox chemistry [80,81]. Finally, Native redox-exfoliated (NRE) MoS_2_ was generated through a redox-driven exfoliation mechanism that leverages naturally occurring surface oxides in MoS_2_ powder. Under appropriate solution-processing conditions (bath sonication), these surface oxides facilitate exfoliation without the use of external redox agents.

Although all samples were ultimately converted to the 1/2H phase, the resulting surface properties and electronic configurations differed substantially due to exfoliation-induced effects. Thin films (approximately 50 nm thick) fabricated from these dispersions exhibited marked differences in optical constants, including refractive indices (*n*) and extinction coefficients (*k*), reflecting variations in chemical composition and electronic structure. Among the samples, NRE MoS_2_ exhibited the highest electron mobility, outperforming RE, SME, and CEPR MoS_2_. Chemical analysis suggested distinct stabilization mechanisms and varying levels of post-exfoliation residues, including POMs and other stabilizing species that were not completely removed during washing steps.

**Figure 14:**
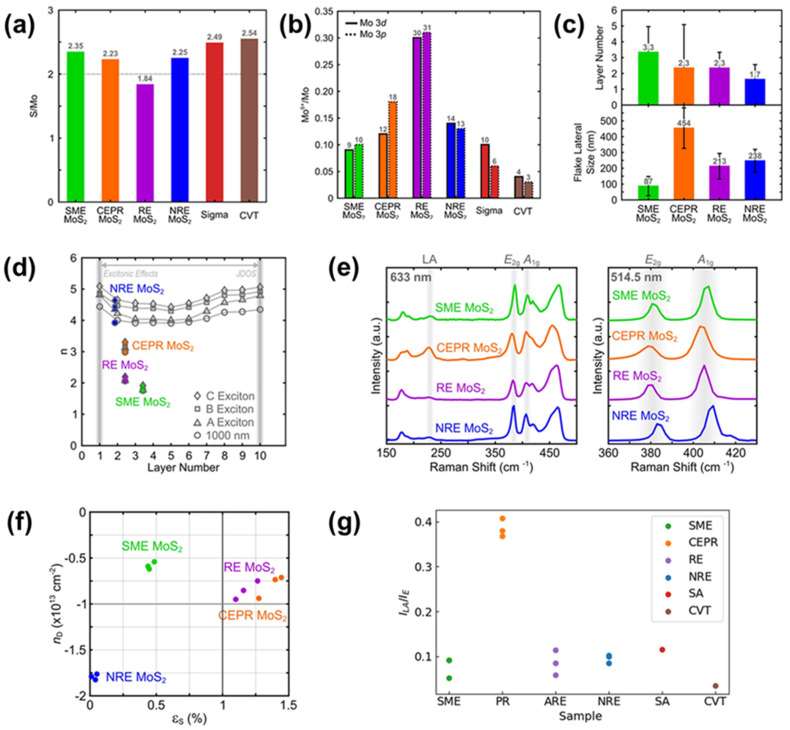
XPS analysis of exfoliation procedure-dependent MoS_2_: (**a**) S/Mo ratios derived from fitted Mo 3p and S 2p peaks and (**b**) Mo^6+^/Mo atomic percentages obtained from fitted Mo 3d and S 2p peaks (solid outline) and from Mo 3p and S 2p peaks (dashed outline). (**c**) Dimensions of exfoliation procedure-dependent MoS_2_ flakes: (top) number of layers and (bottom) lateral flake size. (**d**) Refractive indices (*n*) derived from A, B, and C excitonic energies and at 1000 nm for each MoS_2_ thin film, plotted as a function of the corresponding MoS_2_ flake thickness. (**e**) Resonant (λ_exc_ = 633 nm) and non-resonant (λ_exc_ = 514.15 nm) Raman spectra of each exfoliation procedure-dependent MoS_2_ thin film. (**f**) Carrier doping and intra-flake lattice strain of exfoliation procedure-dependent MoS_2_. (**g**) LA/E^1^_2g_ intensity ratios for each exfoliated MoS_2_ thin film and powder source (MoS_2_ powder from Sigma Aldrich is denoted as SA) [31]. Reprinted in part with permission from Ref. [31]. Copyright 2023, Springer Nature Ltd.

The S/Mo ratios of NRE, SME, and CEPR MoS_2_ were all greater than 2 but lower than that of initial CVT MoS_2_ powder (~2.54), suggesting that the pristine material might have contained excess sulfur, consistent with recently reported surface impurities [95]. In contrast, the S/Mo ratio of RE MoS_2_ was below 2, consistent with the extensive oxidation–reduction cycles involved in its preparation (Figure 14). These compositional trends were also reflected in surface chemistry. RE MoS_2_ exhibited the highest Mo^6+^/Mo ratio, indicating presence larger fraction of molybdenum oxide species, likely originating from adsorption of redox by-products that interact strongly with defect sites in the lattice [83]. In addition, defect concentration and distribution varied across the samples depending on the exfoliation method, although accurate quantification remains challenging due to the limitations of XPS and the polydispersity of exfoliated flakes. Overall, the combined effects of electron-withdrawing POMs, residual by-products, and processing conditions collectively modulate the electronic and optical properties of exfoliated materials.

Given the structural and chemical complexity of these dispersions, Raman spectroscopic analysis should be interpreted as reflecting the cumulative effects of both doping and intra-flake lattice strain. These contributions might be tentatively deconvoluted by estimating the dopant concentration and strain percentage (Figure 14). In addition, under resonant excitation conditions, the intensity ratio between the LA mode (associated with scattering of longitudinal acoustic phonons at the M point of the Brillouin zone) and either the E^1^_2g_ or A_1g_ mode provides a useful metric for assessing lattice disorder and defect density in exfoliated Group VI LTMDs.

Nonetheless, it is important to emphasize that quantitative estimation of carrier doping and intra-flake lattice strain remains tentative due to several technical limitations. The strain (*ε_s_*) and carrier doping concentration (*n_D_*) are typically estimated using Equations (1) and (2) below, where Δ*ω_E_* and Δ*ω_A_* represent the Raman shifts in the E^1^_2g_ and A_1g_ modes, respectively. The Grüneisen parameters (*γ*) and the coefficients *k_n_* are constants relating changes in lattice volume and dopant concentration to vibrational frequency shifts. The reference frequencies $\omega_{E}^{o}$ and $\omega_{A}^{o}$, corresponding to undoped and unstrained MoS_2_, are generally approximated using averaged values due to the polydispersity of dispersions (Figure 14).

(1) $$\Delta \omega_{E}=-2\gamma_{E}\omega_{E}^{o}\varepsilon_{S}+k_{n,E}n_{D}$$

(2) $$\Delta \omega_{A}=-2\gamma_{A}\omega_{A}^{o}\varepsilon_{S}+k_{n,A}n_{D}$$

However, the values of *k*_*n*,*E*_ and *k*_*n*,*A*_ are typically derived exclusively from MoS_2_ monolayers, since corresponding parameters for multilayer systems remain unavailable. Similarly, *γ_E_* and *γ_A_* values are often adopted from data reported for few-layer MoS_2_. In addition, the use of LA/E^1^_2g_ or LA/A_1g_ intensity ratios as indicators of defect density relies on several assumptions. Prior studies have shown that the slopes of LA/E^1^_2g_ and LA/A_1g_ ratios as a function of inter-defect distance are comparable [172], suggesting that relative E^1^_2g_/A_1g_ intensity ratios might serve as a proxy for comparing defect density. Nevertheless, the E^1^_2g_/A_1g_ ratio is sensitive to flake orientation, particularly whether the probe region lies on the basal plane or near an edge [173]. Therefore, comparative analysis generally assumes that flake orientation and film uniformity are approximately consistent across samples.

##### Group VI LTMD–Ligand Hybrids

In hybridized Group VI LTMD–ligand systems, application of the aforementioned model becomes increasingly complex. In most cases, exfoliated dispersions exhibit substantial variability in flake thickness, which contributes to the structural heterogeneity of the resulting hybrids. In addition, surface ligand coverage depends strongly on both the functionalization strategy and the chemical structure of the ligand. While chemical passivation at chalcogen vacancies or covalent linkages at edge sites typically induces localized effects with minimal influence on the lattice structure [124,126], uniform basal-plane functionalization can impose significant structural perturbations. It should also be noted that lattice modifications observed in heavily treated samples often arise from defect formation induced by aggressive chemical conditions rather than from functionalization itself [174,175].

The most suitable systems for application of Equations (1) and (2) above are intercalated structures composed predominantly of LTMD monolayers. Although achieving a fully monolayer dispersion is not feasible, monolayer-enriched fractions can be obtained through cascade centrifugation, albeit at the expense of flake size. This trade-off influences several aspects of intercalated hybrids, which are discussed in Section 5.3. In the present discussion, only thickness effects are considered for simplification, assuming an idealized monodisperse system and neglecting additional variables such as flake size, surfactants, and residual stabilizers.

In general, when a substantial fraction of LTMD monolayer surfaces is covered by ligands, intercalated structures characterized by expanded interlayer spacing are expected to form. In such systems, monolayers are effectively separated by organic guest layers and might retain monolayer-like properties, as reported in several studies [10,55,87,129]. Among the most extensively studied examples are intercalated MoS_2_ superlattices, in which the Raman peak separation between the E^1^_2g_ and A_1g_ modes exceeds 19 cm^−1^, the typical value for intrinsic MoS_2_ monolayers, and approaches ~25 cm^−1^, which is characteristic of bulk MoS_2_ [100,115,116]. This observation suggests a combined influence of doping and lattice strain, raising an important question regarding the appropriate choice of reference frequencies ($\omega_{E}^{o}$ and $\omega_{A}^{o}$) for Raman-based doping and strain analysis. Specifically, it remains unclear whether $\omega_{E}^{o}$ and $\omega_{A}^{o}$ should be defined using monolayer or multilayer values when the system consists of restacked intercalated structures. This consideration is critical for accurate interpretation of Raman-derived doping and strain values in structurally complex hybrid systems.

### 5.3. The Impact of Heterogeneity

Heterogeneity remains a major challenge in all solution-based exfoliation techniques, leading to inherently heterogeneous dispersions. These dispersions can generally be described by three interdependent parameters: thickness, flake size (lateral length and width), and surface charge. Since heterogeneity in exfoliated dispersions was briefly discussed in Section 3.6, this section focuses on the implications of heterogeneity in hybrid structures.

#### 5.3.1. Thickness

A broad thickness distribution is unavoidable in solution-processed 2D dispersions. This parameter, together with effects introduced by specific exfoliation methods, strongly influences the optical properties of exfoliated materials. For example, thickness directly affects the refractive indices of the resulting thin films (Figure 14). Inadequate isolation of monolayer-enrich fractions might therefore lead to misinterpretation of experimental data and potentially incorrect conclusions.

When organic ligands bind robustly to the surfaces of LTMD flakes through ionic or covalent interactions, expansion of the interlayer spacing is expected. This structural change can be detected by X-ray diffraction (XRD), typically through the emergence of low-angle reflection peaks. Since XRD reflections arise from periodic ordering, the formation of an intercalated structure requires a regular arrangement of monolayers separated by organic intercalants. In contrast, mixtures of few-layer to monolayer flakes can restack into multiple configurations. Except for the case of periodically stacked monolayers, these structures often resemble unintercalated multilayer materials in their XRD patterns [100]. Furthermore, random restacking during self-assembly or sample preparation might yield inconsistent structural information, complicating data interpretation. For example, in the study by Pérez and co-workers, evidence supported basal-plane functionalization; however, the absence of low-angle diffraction peaks suggested a mixed distribution of few-layer and monolayer flakes (mean flake thickness ~12 nm) [176]. In contrast, monolayer-enriched dispersions consistently yield observable intercalated structures across XRD measurements [100].

Beyond structural characterization, XRD results might provide insight into the mechanism driving LTMD–ligand hybrid formation. Assembly of intercalated superlattice structures from functionalized flakes might proceed either through a true intercalation mechanism or exclusively through surface functionalization followed by restacking. In the case of intercalation, the extent of intercalation depends on critical experimental parameters such as concentration, temperature, and reaction time [121]. If ligand binding is restricted to surface functionalization, a fully intercalated state might remain inaccessible regardless of experimental conditions [100].

#### 5.3.2. Flake Size

In solution-processed dispersions, the size and morphology of exfoliated 2D flakes cannot be precisely controlled. As a result, flake area cannot be directly measured and is typically estimated using assumptions based on maximum lateral length and width. Flake surface area strongly influences ligand surface coverage and the resulting structural organization of the organic environment. This dependence arises from multiple factors, including vdW dimensions, interchain interactions, and ligand orientation. However, XRD cannot directly provide information on ligand packing density, molecular orientation, or phase behavior within the organic environment.

**Figure 15:**
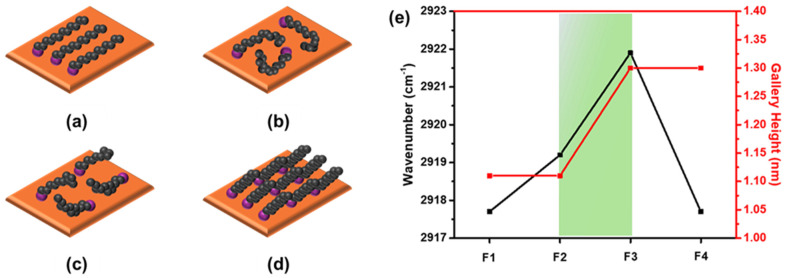
(**a**–**d**) Schematic illustration of structural variations in self-assembled monolayers (SAMs) extending from both sides of an LTMD flake within the interlayer galleries of restacked superlattice structures. Panels (**a**–**d**) correspond to hybrids prepared from MoS_2_ fractions collected at centrifugation rates of 2000–2500, 2500–3000, 3000–4000, and 4000–10,000 rpm, denoted as F1–F4, respectively. From F1 to F4, the average flake size decreases. (**e**) *ν_as_*^(CH2)^ frequencies and gallery heights of NHC/MoS_2_ hybrids as a function of flake area, measured at room temperature. The green region denotes highly disordered phases. Reprinted in part with permission from Ref. [100]. Copyright 2025, American Chemical Society.

Vaia and co-workers reported that infrared (IR) spectroscopy can be used to extract structural information related to molecular ordering, particularly for ligands containing hydrocarbon chains [177]. Building on this approach, our recent work showed that the organic environments within the interlayer galleries of intercalated MoS_2_ exhibit distinct structural characteristics that depend on flake area. By monitoring the asymmetric methylene stretching vibration (*ν_as_*^(CH2)^), a preliminary assessment of molecular ordering in SAMs on LTMD surfaces was obtained. Due to the interchain interactions and variations in flake area, ligands tended to maximize packing density, which induced conformational disorder and formation of kinks along hydrocarbon chains. These structural perturbations gave to measurable shifts in the *ν_as_*^(CH2)^ mode (Figure 15). This observation highlights the diversity of structural phases not only within periodically stacked monolayer superlattices but also among mixed few-layer and monolayer configurations.

In addition, ligand packing effects might contribute to variations in doping levels, intra-flake strain, and lattice disorder. Although interference effects from multilayer flakes are unavoidable in solution-processed samples, such structurally induced variations can be identified through systematic trends in vibrational modes and correlated changes in the optical band gap, as reflected in PL emission spectra [100].

**Figure 16:**
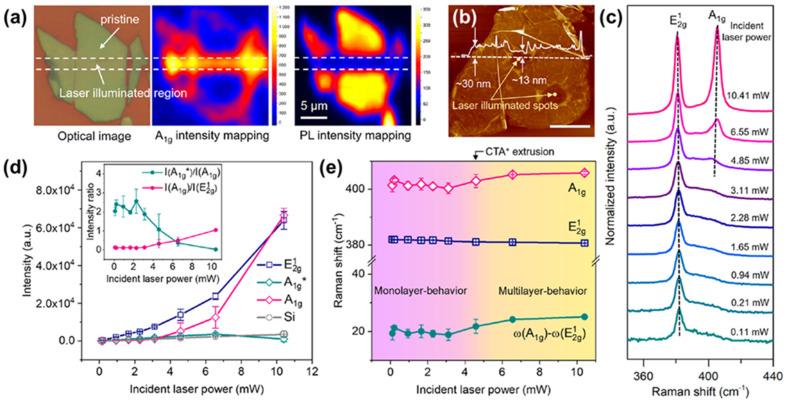
Influence of incident laser power on Raman spectra. (**a**) Optical image, A_1g_ intensity mapping, and PL intensity mapping of a CTA^+^-intercalated MoS_2_ flake treated with high-power laser along the dashed lines. (**b**) AFM morphology of the CTA^+^–MoS_2_ thin flakes after high-power laser treatment. The solid line shows the height profile along the dashed line (scale bar: 5 μm). (**c**) Evolution of Raman spectra with increasing incident laser power. (**d**) Evolution of the intensities of the E_2g_^1^, A_1g_*, and A_1g_ peaks with increasing incident laser power. The inset shows the evolution of the intensity ratios of I(A_1g_*)/I(A_1g_) and I(A_1g_)/I(E_2g_^1^). Peak intensities and peak positions were obtained from fits to the Raman spectra. (**e**) Evolution of the peak positions and peak separation between the E_2g_^1^ and A_1g_ modes with increasing incident laser power. Reprinted in part with permission from Ref. [87]. Copyright 2023, American Chemical Society.

The investigation of organic environments within intercalated Group VI LTMDs remains largely unexplored. At larger flake sizes, where lateral dimensions exceed the micrometer scale, such effects have rarely been reported. Recent studies employing electrochemical intercalation in single-crystal Group VI LTMDs (e.g., MoS_2_ and WSe_2_) suggest that size-dependent effects might be minimized, based on the assumption that precise electrochemical control enables the formation of well-organized SAMs within the interlayer galleries [55,87]. Notably, in CTA^+^-intercalated MoS_2_, an anomalous out-of-plane Raman vibrational mode (A_1g_) was observed. Specifically, the original A_1g_ peak was suppressed and a new peak, denoted A_1g_*, emerged near the position of the E^1^_2g_ mode, which remained essentially unshifted (Figure 16). The authors investigated the laser power dependence of the A_1g_ mode. The irradiated region exhibited bulk-like MoS_2_ behavior, with no detectable PL signal (Figure 16a). As the laser power increased, the interlayer spacing of the intercalated structure reverted to that of the unintercalated state (Figure 16b), and the original A_1g_ peak was restored (Figure 16c–e). This behavior suggested that the intercalants were thermally expelled from the irradiated region due to localized heating, thereby regenerating pristine MoS_2_.

The red shift in the original A_1g_ mode and the emergence of A_1g_* were attributed to strong *n*-doping and lattice strain induced by interactions between CTA^+^ and the MoS_2_ lattice. This observation suggests that the out-of-plane A_1g_ mode, in addition to the in-plane E^1^_2g_ mode, might serve as a sensitive indicator of surface strain in large-area flakes—an effect that is not typically observed in flakes with nanometer-scale lateral dimensions. However, IR spectroscopy was not performed to evaluate the structural ordering of the organic intercalants. This omission raises an important question: if SAMs formed within the interlayer galleries of large-area monolayers are highly ordered, do they exert minimal perturbation on the intralayer lattice? More broadly, considerate remains unclear whether strain inferred from E^1^_2g_ shifts in other intercalated systems reflects localized perturbations rather than uniform lattice distortion.

#### 5.3.3. Surface Charge–Stabilization Mechanism

In hybrid LTMD–ligand systems, the colloidal state can be maintained when the attached ligands establish favorable solvent–ligand interactions with the dispersion medium [130]. A critical concern, however, is whether adsorbed surface species, such as by-products originating from exfoliation processes, interfere with ligand functionalization and thereby influence the achievable surface coverage. This issue is particularly relevant because restacked structures, whether intercalated or unintercalated, are often kinetically controlled.

In systems where strong LTMD–ligand interactions are absent, leading to an unintercalated configuration, the underlying cause is often disruption of the original stabilization mechanism. In some cases, this destabilization can dominate over the functionalization process, further limiting ligand attachment and colloidal stability. For example, in Group VI LTMD flakes (MoS_2_ and WS_2_), desorption of surface-bound POMs, followed by attachment of masked NHCs, was shown to mitigate destabilization. This sequential process facilitated the formation of well-ordered SAMs within the interlayer galleries. In contrast, MoS_2_ dispersions prepared via a surface/solvent matching approach exhibited an unintercalated state after the first exposure to masked NHCs [100]. An intercalated state was achieved only after a second masked NHC treatment combined with brief sonication to redisperse the restacked monolayers, indicating that additional processing steps were required to overcome kinetic barriers to intercalation. Furthermore, rapid restacking led to the formation of highly disordered SAMs and reduced packing density of the organic layers relative to systems containing surface-adsorbed POMs [100]. Nevertheless, surface-adsorbed species might also hinder complete intercalation by obstructing ligand penetration into the interlayer galleries, as observed in Group V LTMDs [121]. These observations highlight the critical role of surface-anchored species in regulating the structural organization, packing density, and stability of intercalated SAMs in LTMD–ligand hybrid systems.

## 6. Conclusions and Outlook

In this review, we address the diversity in the properties of exfoliated Group VI LTMD nanosheets arising from the wide range of solution-processing techniques reported to date. Key factors including the electron density, doping, and surface properties of exfoliated LTMD dispersions were discussed and categorized into distinct classes. These exfoliation-dependent properties are expected to play a central role in guiding strategies for surface functionalization. Contrary to common assumptions, an unexplored significant gap remains in the types of ligands employed to functionalize specific classes of exfoliated Group VI LTMD dispersions. Building on nucleophile–electrophile interactions and Lewis acid–base principles, we propose that systemic investigation of these gaps might enable the development of new Group VI LTMD–ligand hybrid systems.

A persistent challenge lies in the inherent heterogeneity of dispersions produced by solution-based exfoliation methods. This heterogeneity includes variations in crystallographic phase composition, electron density, surface charge, and flake dimensions such as thickness and lateral size, all of which complicate interpretation in experimental data for both exfoliated materials and their hybrids. In addition, recent studies have demonstrated that widely accepted interpretations might not be universally applicable across different systems, even when similar characterization techniques are employed. Herein, we summarized these discrepancies and highlighted the need for caution. Accordingly, the development of improved analytical models and standardized sample preparation protocols for characterization methods, particularly Raman spectroscopy, is necessary to enable consistent and reliable comparisons across studies.

Finally, the quality of exfoliated nanosheets is strongly dependent on the quality of the bulk starting materials. In addition, the monolayer content within dispersions plays a critical role in elucidating LTMD–ligand interaction mechanisms and determining the final structure and properties of LTMD–ligand hybrids. These outcomes are further influenced by flake size. However, as discussed throughout this review, flake size and thickness are interdependent parameters, highlighting the need for strategies that enable the production of monolayer-enriched dispersions with controllable monolayer flake dimensions.

## Figures and Tables

**Table 1:** Summary of properties of exfoliated Group VI LTMD dispersions obtained via various solution-processed exfoliation methods.

Exfoliation Method	Phase	Doping State	Surface Charge	Surfactant	Ref.
**Intercalation**					
Li intercalation	1T	*n*-doped	Negative	Intercalants	[22,50]
Li intercalation	1/2H	*n*-doped	Negative	Intercalants	[22]
Li intercalation	Mixture	*n*-doped	Negative	Intercalants	[16,19,21,22]
Other cation intercalation	Mixture	*n*-doped	Negative	Intercalants	[51,84]
Anion intercalation	1/2H	*p*-doped	Negative	Redox species	[47]
Molecular intercalation	1T	*n*-doped	Negative	Intercalants	[55,85,86]
Molecular intercalation	1/2H	*n*-doped	Negative	Intercalants	[54,55,56,87]
**Surface matching—sonication mediated**					
Surface/solvent matching	1/2H	Undoped	Intrinsic	None	[58,63]
Surfactant-assisted (SDS/SC)	1/2H	Undoped	Negative	SDS/SC	[64,65,68,88]
Surfactant-assisted (CTAB)	1/2H	Undoped	Positive	CTAB	[68]
Polymer-assisted	1/2H	Undoped	Not specified	PVA/BSA/PAA/PVP	[66,67]
Mild reductant-treated (NaBH_4_)	1/2H	*n*-doped	Negative	None	[23]
Salt-assisted	1/2H	Undoped	Not specified	Intercalants	[71]
Solvent grinding	1/2H	Undoped	Not specified	None	[72,73]
**Mega-sonication**	1/2H	*n*-doped	Negative	None	[89,90]
**Microwave-assisted**	1/2H	Undoped	Intrinsic	None	[77,78]
**Redox chemistry**	1/2H	*p*-doped	Negative	Redox species (POMs)	[57,80,81]

**Abbreviations:** sodium dodecyl sulfate (SDS), cetyltrimethylammonium bromide (CTAB), sodium cholate (SC), poly(vinyl alcohol) (PVA), bovine serum albumin (BSA), poly(acrylic acid) (PAA), and poly(vinyl pyrrolidone) (PVP).

**Table 2:** Summary of E^1^_2g_ and A_1g_ Raman peak positions and their separation in Group VI LMTDs for monolayer and multilayer architectures.

Group VI LTMDs	E^1^_2g_ (cm^−1^)	A_1g_ (cm^−1^)	Peak Separation (cm^−1^)	Ref.
Monolayer MoS_2_	384.6	403.4	18.8	[115,116]
Multilayer MoS_2_	383.0	408.0	25.0	
Monolayer MoSe_2_	240.5	287.2	46.7	[119]
Multilayer MoSe_2_	242.5	286.0	43.5	
Monolayer WS_2_	356.0	417.5	61.5	[120]
Multilayer WS_2_	355.5	420.5	65.0	
Monolayer WSe_2_	~250.0	~250.0	N/A	[119]
Multilayer WSe_2_	248.0	250.8	2.8	

## Data Availability

No new data were created or analyzed in this study.

## Supplementary Materials

The following supporting information can be downloaded at: https://www.mdpi.com/article/10.3390/nano16070429/s1, Table S1: MoS_2_ powder sources and their corresponding elemental compositions; The table is reproduced with permission from ref. [94]. Copyright 2019, American Chemical Society; Table S2: Summary of exfoliated Group VI LTMD flake size and thickness obtained via various solution-processed exfoliation methods; Table S3: Summary of characterization techniques for exfoliated dispersions of 2D materials.
