# Supplementary material for: Interplay Between Exfoliation and Functionalization Strategies for Group VI Layered Transition Metal Dichalcogenide Dispersions
Source: Nanomaterials (Basel). 2026 Mar 31;16(7):429. doi: 10.3390/nano16070429 (PMC13074869; doi:10.3390/nano16070429)
Supplement: Supplementary file 1 [file nanomaterials-16-00429-s001.zip › nanomaterials-4196251-supplementary.pdf]

# Supporting Information

# Interplay Between Exfoliation and Functionalization Strategies for Group VI Layered Transition Metal Dichalcogenide Dispersions

**Quoc Minh Tran, Pailinrut Chinwangso, Minh Dang Nguyen, Supawitch Hoijang, Melissa Ariza Gonzalez, Ruwanthi Amarasekara, Ramtin Yarinia, Yunsoo Choi and T. Randall Lee ***

Department of Chemistry and the Texas Center for Superconductivity, University of Houston, 4800 Calhoun Road, Houston, Texas 77204-5003, United States
* Correspondence: trlee@uh.edu

**Table S1.** $MoS_2$ powder sources and their corresponding elemental compositions. The table is reproduced with permission from ref 1. Copyright 2019, American Chemical Society.

| Starting Material | Label | Mo:S Ratio |
|---|---|---|
| Sigma (90 nm) | SAnp | 1 : (2.11 ± 0.00) |
| Sigma (2 μm) | SA2 | 1 : (1.92 ± 0.00) |
| Sigma (6 μm) | SA6 | 1 : (1.98 ± 0.03) |
| Alfa-Aesar (2 μm) | AA | 1 : (1.77 ± 0.30) |
| Tribotecc | Tribo | 1 : (1.78 ± 0.07) |
| SPI Supplies | Crystal | 1 : (2.16 ± 0.19) |

**Table S2.** Summary of exfoliated Group VI LTMD flake size and thickness obtained via various solution-processed exfoliation methods.

| Exfoliation Method | Lateral Size | Number of Layer | Comments | Ref. |
|---|---|---|---|---|
| **Intercalation** | | | | |
| Li intercalation (chemical) | <1.5 μm | 1–5 | 1T(−) / *n*-1/2H(−) | 2,3 |
| Li intercalation (electrochemical) | <1 μm | 1–5 | 1T(−) | 4,5 |
| Othe cation intercalation | 1–10 μm | 1–2 | 1T(−) / *n*-1/2H(−) | 6,7 |
| Anion intercalation | Up to 50 μm | 1–2 | *p*-1/2H(−) | 8 |
| Molecular intercalation | 0.5–2 μm | 1–4 | 1T(−) / *n*-1/2H(−) | 9–11 |
| **Surface Matching** | | | | |
| Surface/solvent matching | < 0.5 μm | 1–20 | 1/2H | 12–16 |
| Surfactant | < 0.5 μm | 1–20 | 1/2H(+) / 1/2H(−) | 17–22 |
| Mild reductant-treated ($NaBH_4$) | N/A | N/A | *n*-1/2H(−) | 23 |
| Salt-assisted | < 1 μm | 1–5 | 1/2H | 24 |
| Solvent grinding | < 0.3 μm | 2–5 | 1/2H | 25,26 |
| Pre-expansion | > 0.5 μm | 5–6 | 1/2H | 27,28 |
| **Mega-sonication** | 0.5–1 μm | 1–2 | *n*-1/2H(−) | 29,30 |
| **Microwave** | > 1 μm | 1–4 | 1/2H | 31–33 |
| **Redox chemistry** | < 0.5 μm | 1–2 | *p*-1/2H(−) | 34,35 |

**Table S3.** Summary of characterization techniques for exfoliated dispersions of 2D materials .

| **Characterization Techniques** | | | |
|---|---|---|---|
| **Structure Information** | | | |
| Scanning electron microscopy | SEM | Solid | Based on the backscattered secondary electrons from sample<br>Morphology of material with nm resolution |
| Transmission electron microscopy | TEM | Solid | Based on the transmitted electrons through sample |

| | | | |
|---|---|---|---|
| Atomic force microscopy | AFM | Solid | Morphology of material with resolution down to the atomic level<br>Based on the repulsive force between the tip and sample surface<br>Topographic images of surface, thickness of 2D materials |
| *X*-ray diffraction | XRD | Solid/Liquid | Based on the scattering of *X*-rays by the atoms of sample<br>Crystal structure of material, including:<br>• Interlayer distance<br>• Average crystalline size (number of layers in 2D materials) |
| Raman spectroscopy | Raman | Solid | Based on the inelastic scattering of photons due to specific vibrations<br>Number of layers, defects, doping effects, degree of strain, *etc*. |
| **Electronic Properties** | | | |
| *X*-ray photoelectron spectroscopy | XPS | Solid | Based on the interactions of *X*-ray with sample<br>Electronic state/chemical environment of elements<br>Band structure of materials in valence band region |
| Ultraviolet-visible spectroscopy | UV–Vis | Liquid | Based on the absorption, reflection and scattering of light in interactions with sample<br>Excitonic transitions, tentative information about size of materials |
| Photoluminescence spectroscopy | PL | Solid/Liquid | Based on the emission of photons during electronic relaxation to the ground state<br>Excitonic transitions/Band gap structure of materials |
| Ultraviolet photoelectron spectroscopy | UPS | Solid | Based on the interactions of UV photons with sample<br>Band gap structure of materials |
| **Chemical Composition** | | | |
| *X*-ray photoelectron spectroscopy | XPS | Solid | Based on the interactions of *X*-ray with sample<br>The elemental ratio corresponding to similar or different electronic states is determined based on the peak area analysis |
| **Surface Characteristics** | | | |
| Zeta Potentials | Zeta | Liquid | Based on the electrochemical equilibrium at the material-liquid interface<br>Surface charges of material in solution |

## References


(1) Ott, S.; Wolff, N.; Rashvand, F.; Rao, V. J.; Zaumseil, J.; Backes, C. Impact of the $MoS_2$ Starting Material on the Dispersion Quality and Quantity after Liquid Phase Exfoliation. *Chem. Mater.* **2019**, *31*, 8424–8431.

(2) Fan, X.; Xu, P.; Li, Y. C.; Zhou, D.; Sun, Y.; Nguyen, M. A. T.; Terrones, M.; Mallouk, T. E. Controlled Exfoliation of $MoS_2$ Crystals into Trilayer Nanosheets. *J. Am. Chem. Soc.* **2016**, *138*, 5143–5149.

(3) Fan, X.; Xu, P.; Zhou, D.; Sun, Y.; Li, Y. C.; Nguyen, M. A. T.; Terrones, M.; Mallouk, T. E. Fast and Efficient Preparation of Exfoliated 2H $MoS_2$ Nanosheets by Sonication-Assisted Lithium Intercalation and Infrared Laser-Induced 1T to 2H Phase Reversion. *Nano Lett.* **2015**, *15*, 5956–5960.

(4) Zhu, X.; Su, Z.; Wu, C.; Cong, H.; Ai, X.; Yang, H.; Qian, J. Exfoliation of $MoS_2$ Nanosheets Enabled by a Redox-Potential-Matched Chemical Lithiation Reaction. *Nano Lett.* **2022**, *22*, 2956–2963.

(5) Yang, R.; Mei, L.; Zhang, Q.; Fan, Y.; Shin, H. S.; Voiry, D.; Zeng, Z. High-Yield Production of Mono- or Few-Layer Transition Metal Dichalcogenide Nanosheets by an Electrochemical Lithium Ion Intercalation-Based Exfoliation Method. *Nat Protoc* **2022**, *17*, 358–377.

(6) Zheng, J.; Zhang, H.; Dong, S.; Liu, Y.; Tai Nai, C.; Suk Shin, H.; Young Jeong, H.; Liu, B.; Ping Loh, K. High Yield Exfoliation of Two-Dimensional Chalcogenides Using Sodium Naphthalenide. *Nat. Commun.* **2014**, *5*, 2995.

(7) Zhao, D.; Xu, S.; Wang, H.; Shen, Y.; Xu, Q. Exfoliation of $MoS_2$ by Zero-Valent Transition Metal Intercalation. *Chem. Commun.* **2023**, *59*, 8135–8138.

(8) Liu, N.; Kim, P.; Kim, J. H.; Ye, J. H.; Kim, S.; Lee, C. J. Large-Area Atomically Thin $MoS_2$ Nanosheets Prepared Using Electrochemical Exfoliation. *ACS Nano* **2014**, *8*, 6902–6910.

(9) Wang, C.; He, Q.; Halim, U.; Liu, Y.; Zhu, E.; Lin, Z.; Xiao, H.; Duan, X.; Feng, Z.; Cheng, R.; Weiss, N. O.; Ye, G.; Huang, Y.-C.; Wu, H.; Cheng, H.-C.; Shakir, I.; Liao, L.; Chen, X.; Goddard III, W. A.; Huang, Y.; Duan, X. Monolayer Atomic Crystal Molecular Superlattices. *Nature* **2018**, *555*, 231–236.

(10) Zhou, B.; Zhou, J.; Wang, L.; Kang, J. H.; Zhang, A.; Zhou, J.; Zhang, D.; Xu, D.; Hu, B.; Deng, S.; Huang, L.; Wong, C. W.; Huang, Y.; Duan, X. A Chemical-Dedoping Strategy to Tailor Electron Density in Molecular-Intercalated Bulk Monolayer $MoS_2$. *Nat. Synth.* **2024**, *3*, 67–75.

(11) Lin, Z.; Liu, Y.; Halim, U.; Ding, M.; Liu, Y.; Wang, Y.; Jia, C.; Chen, P.; Duan, X.; Wang, C.; Song, F.; Li, M.; Wan, C.; Huang, Y.; Duan, X. Solution-Processable 2D Semiconductors for High-Performance Large-Area Electronics. *Nature* **2018**, *562*, 254–258.

(12) Coleman, J. N.; Lotya, M.; O'Neill, A.; Bergin, S. D.; King, P. J.; Khan, U.; Young, K.; Gaucher, A.; De, S.; Smith, R. J.; Shvets, I. V.; Arora, S. K.; Stanton, G.; Kim, H.-Y.; Lee, K.; Kim, G. T.; Duesberg, G. S.; Hallam, T.; Boland, J. J.; Wang, J. J.; et al. Two-Dimensional Nanosheets Produced by Liquid Exfoliation of Layered Materials. *Science* **2011**, *331*, 568–571.

(13) Shen, J.; Wu, J.; Wang, M.; Dong, P.; Xu, J.; Li, X.; Zhang, X.; Yuan, J.; Wang, X.; Ye, M.; Vajtai, R.; Lou, J.; Ajayan, P. M. Surface Tension Components Based Selection of Cosolvents for Efficient Liquid Phase Exfoliation of 2D Materials. *Small* **2016**, *12*, 2741–2749.

(14) Wang, M.; Xu, X.; Ge, Y.; Dong, P.; Baines, R.; Ajayan, P. M.; Ye, M.; Shen, J. Surface Tension Components Ratio: An Efficient Parameter for Direct Liquid Phase Exfoliation. *ACS Appl. Mater. Interfaces* **2017**, *9*, 9168–9175.

(15) Shen, J.; He, Y.; Wu, J.; Gao, C.; Keyshar, K.; Zhang, X.; Yang, Y.; Ye, M.; Vajtai, R.; Lou, J.; Ajayan, P. M. Liquid Phase Exfoliation of Two-Dimensional Materials by Directly Probing and Matching Surface Tension Components. *Nano Lett.* **2015**, *15*, 5449–5454.

(16) Halim, U.; Zheng, C. R.; Chen, Y.; Lin, Z.; Jiang, S.; Cheng, R.; Huang, Y.; Duan, X. A Rational Design of Cosolvent Exfoliation of Layered Materials by Directly Probing Liquid–Solid Interaction. *Nat Commun* **2013**, *4*, 2213.

(17) Gupta, A.; Arunachalam, V.; Vasudevan, S. Water Dispersible, Positively and Negatively Charged $MoS_2$ Nanosheets: Surface Chemistry and the Role of Surfactant Binding. *J. Phys. Chem. Lett.* **2015**, *6*, 739–744.

(18) Smith, R. J.; King, P. J.; Lotya, M.; Wirtz, C.; Khan, U.; De, S.; O'Neill, A.; Duesberg, G. S.; Grunlan, J. C.; Moriarty, G.; Chen, J.; Wang, J.; Minett, A. I.; Nicolosi, V.; Coleman, J. N. Large-Scale Exfoliation of Inorganic Layered Compounds in Aqueous Surfactant Solutions. *Advanced Materials* **2011**, *23*, 3944–3948.

(19) Varrla, E.; Backes, C.; Paton, K. R.; Harvey, A.; Gholamvand, Z.; McCauley, J.; Coleman, J. N. Large-Scale Production of Size-Controlled $MoS_2$ Nanosheets by Shear Exfoliation. *Chem. Mater.* **2015**, *27*, 1129–1139.

(20) Backes, C.; Szydłowska, B. M.; Harvey, A.; Yuan, S.; Vega-Mayoral, V.; Davies, B. R.; Zhao, P.; Hanlon, D.; Santos, E. J. G.; Katsnelson, M. I.; Blau, W. J.; Gadermaier, C.; Coleman, J. N. Production of Highly Monolayer Enriched Dispersions of Liquid-Exfoliated Nanosheets by Liquid Cascade Centrifugation. *ACS Nano* **2016**, *10*, 1589–1601.

(21) Vega-Mayoral, V.; Backes, C.; Hanlon, D.; Khan, U.; Gholamvand, Z.; O'Brien, M.; Duesberg, G. S.; Gadermaier, C.; Coleman, J. N. Photoluminescence from Liquid-Exfoliated $WS_2$ Monomers in Poly(Vinyl Alcohol) Polymer Composites. *Advanced Functional Materials* **2016**, *26*, 1028–1039.

(22) Guan, G.; Zhang, S.; Liu, S.; Cai, Y.; Low, M.; Teng, C. P.; Phang, I. Y.; Cheng, Y.; Duei, K. L.; Srinivasan, B. M.; Zheng, Y.; Zhang, Y.-W.; Han, M.-Y. Protein Induces Layer-by-Layer Exfoliation of Transition Metal Dichalcogenides. *J. Am. Chem. Soc.* **2015**, *137* (19), 6152–6155.

(23) Krajewska, A. M.; Paiva, A. E.; Morris, M.; McDonald, A. R. Reduction of Exfoliated $MoS_2$ Nanosheets Yields the Semi-Conducting 2H-Polymorph Rather Than the Metallic 1T-Polymorph. *Eur. J. Inorg. Chem.* **2024**, *27*, e202400292.

(24) Niu, L.; Li, K.; Zhen, H.; Chui, Y.-S.; Zhang, W.; Yan, F.; Zheng, Z. Salt-Assisted High-Throughput Synthesis of Single- and Few-Layer Transition Metal Dichalcogenides and Their Application in Organic Solar Cells. *Small* **2014**, *10*, 4651–4657.

(25) Carey, B. J.; Daeneke, T.; Nguyen, E. P.; Wang, Y.; Ou, J. Z.; Zhuiykov, S.; Kalantar-zadeh, K. Two Solvent Grinding Sonication Method for the Synthesis of Two-Dimensional Tungsten Disulphide Flakes. *Chem. Commun.* **2015**, *51*, 3770–3773.

(26) Nguyen, E. P.; Carey, B. J.; Daeneke, T.; Ou, J. Z.; Latham, K.; Zhuiykov, S.; Kalantar-zadeh, K. Investigation of Two-Solvent Grinding-Assisted Liquid Phase Exfoliation of Layered $MoS_2$. *Chem. Mater.* **2015**, *27*, 53–59.

(27) Pagona, G.; Bittencourt, C.; Arenal, R.; Tagmatarchis, N. Exfoliated Semiconducting Pure 2H-$MoS_2$ and 2H-$WS_2$ Assisted by Chlorosulfonic Acid. *Chem. Commun.* **2015**, *51*, 12950–12953.

(28) Lin, H.; Wang, J.; Luo, Q.; Peng, H.; Luo, C.; Qi, R.; Huang, R.; Travas-Sejdic, J.; Duan, C.-G. Rapid and Highly Efficient Chemical Exfoliation of Layered $MoS_2$ and $WS_2$. *Journal of Alloys and Compounds* **2017**, *699*, 222–229.

(29) Rangnekar, S. V.; Sangwan, V. K.; Jin, M.; Khalaj, M.; Szydłowska, B. M.; Dasgupta, A.; Kuo, L.; Kurtz, H. E.; Marks, T. J.; Hersam, M. C. Electroluminescence from Megasonically Solution-Processed $MoS_2$ Nanosheet Films. *ACS Nano* **2023**, *17*, 17516–17526.

(30) Kuo, L.; Sangwan, V. K.; Rangnekar, S. V.; Chu, T.-C.; Lam, D.; Zhu, Z.; Richter, L. J.; Li, R.; Szydłowska, B. M.; Downing, J. R.; Luijten, B. J.; Lauhon, L. J.; Hersam, M. C. All-Printed Ultrahigh-Responsivity $MoS_2$ Nanosheet Photodetectors Enabled by Megasonic Exfoliation. *Advanced Materials* **2022**, *34*, 2203772.

(31) Wu, W.; Xu, J.; Tang, X.; Xie, P.; Liu, X.; Xu, J.; Zhou, H.; Zhang, D.; Fan, T. Two-Dimensional Nanosheets by Rapid and Efficient Microwave Exfoliation of Layered Materials. *Chem. Mater.* **2018**, *30*, 5932–5940.

(32) Quirós-Ovies, R.; Laborda, M.; Sabanés, N. M.; Martín-Pérez, L.; Silva, S. M.-D.; Burzurí, E.; Sebastian, V.; Pérez, E. M.; Santamaría, J. Microwave-Driven Exfoliation of Bulk 2H-$MoS_2$ after Acetonitrile Prewetting Produces Large-Area Ultrathin Flakes with Exceptionally High Yield. *ACS Nano* **2023**, *17*, 5984–5993.

(33) Liu, Z.; Wang, Y.; Wang, Z.; Yao, Y.; Dai, J.; Das, S.; Hu, L. Solvo-Thermal Microwave-Powered Two-Dimensional Material Exfoliation. *Chem. Commun.* **2016**, *52*, 5757–5760.

(34) Jawaid, A. M.; Ritter, A. J.; Vaia, R. A. Mechanism for Redox Exfoliation of Layered Transition Metal Dichalcogenides. *Chem. Mater.* **2020**, *32*, 6550–6565.

(35) Jawaid, A.; Che, J.; Drummy, L. F.; Bultman, J.; Waite, A.; Hsiao, M.-S.; Vaia, R. A. Redox Exfoliation of Layered Transition Metal Dichalcogenides. *ACS Nano* **2017**, *11*, 635–646.
